# Genetic characterization of schistosome species from cattle in Côte d’Ivoire

**DOI:** 10.1186/s13071-024-06221-9

**Published:** 2024-03-12

**Authors:** Jennifer Giovanoli Evack, Jules N. Kouadio, Louise Y. Achi, Bassirou Bonfoh, Eliézer K. N’Goran, Jakob Zinsstag, Jürg Utzinger, Oliver Balmer

**Affiliations:** 1 Swiss Tropical and Public Health Institute, Allschwil, Switzerland; 2 University of Basel, Basel, Switzerland; 3 Epidemiology, Biostatistics and Prevention Institute, University of Zurich, Zurich, Switzerland; 4 Unité de Formation et de Recherche Biosciences, Université Félix Houphouët-Boigny, Abidjan, Côte d’Ivoire; 5 Centre Suisse de Recherches Scientifiques en Côte d’Ivoire, Abidjan, Côte d’Ivoire; 6 École de Spécialisation en Elevage et Métiers de la Viande de Bingerville, Abidjan, Côte d’Ivoire

**Keywords:** Cattle, Côte d’Ivoire, Genetic diversity, Genetic structuring, Microsatellites, One Health, *Schistosoma bovis*, *Schistosoma curassoni*, Schistosomiasis

## Abstract

**Background:**

Schistosomiasis is a water-based parasitic disease that affects humans, livestock and wild animals. While considerable resources are dedicated to the surveillance, disease mapping, control and elimination of human schistosomiasis, this is not the case for livestock schistosomiasis. Indeed, there are important data and knowledge gaps concerning the species present, population genetic diversity, infection prevalence, morbidity and economic impact. This study aimed to identify circulating schistosome species in cattle across Côte d’Ivoire and to investigate their population diversity and structuring.

**Methods:**

Overall, 400 adult schistosomes were collected from slaughtered cattle at six sites across Côte d’Ivoire. Additionally, 114 miracidia were collected from live cattle at one site: Ferkessédougou, in the northern part of Côte d’Ivoire. DNA from all specimens was extracted and the *cox1* and *ITS1/2* regions amplified and analysed to confirm species. The genetic diversity and structuring of the schistosome populations were investigated using 12 microsatellite markers.

**Results:**

All adult schistosomes and miracidia presented *Schistosoma bovis* mitochondrial *cox1* profile. Nuclear *ITS1/*2 data were obtained from 101 adult schistosomes and four miracidia, all of which presented an *S. bovis* profile. Genetic diversity indices revealed a deficiency of heterozygotes and signals of inbreeding across all sites, while structure analyses displayed little geographic structuring and differentiation. Cattle in Côte d’Ivoire thus appear to be mono-species infected with *S. bovis*. Hybrids of *Schistosoma haematobium* × *S. bovis* have not been identified in this study. Cattle schistosomes appear to be panmictic across the country.

**Conclusions:**

Our results contribute to a deeper understanding of schistosome populations in Ivorian cattle and emphasize a One Health approach of joint human and animal surveillance and prevention and control programmes for schistosomiasis.

**Graphical Abstract:**

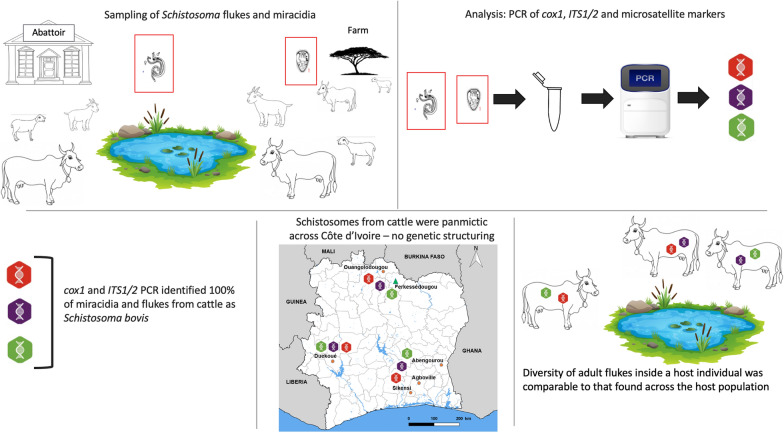

**Supplementary Information:**

The online version contains supplementary material available at 10.1186/s13071-024-06221-9.

## Background

*Schistosoma*, a genus of trematodal parasites, causes schistosomiasis, one of 21 diseases that the World Health Organization (WHO) groups as neglected tropical diseases (NTDs). There are over 20 known species of schistosomes, some of which are zoonotic, infecting a wide range of definitive hosts from livestock and wildlife to humans [1]. Schistosomiasis has a focal distribution, depending on the presence of their respective definitive and intermediate snail hosts in the ecosystem. In humans, an estimated 90% of all schistosomiasis cases occur in sub-Saharan Africa, where there are two main endemic species: *Schistosoma haematobium* (causing urogenital schistosomiasis) and *Schistosoma mansoni* (causing intestinal schistosomiasis) [2]. Thanks to a focus on morbidity control over the past 2 decades, human schistosomiasis is reasonably well mapped and the prevalence of infection among school-aged children has decreased by more than 50% [3, 4]. Periodic mass drug administration, mainly targeting school-aged children, has been the mainstay of control efforts. Yet, hotspots of infection remain, leading to questions of the role of livestock and wildlife in the disease transmission cycle and whether a One Health approach could close the gap to disease elimination [5, 6].

Only few resources are dedicated to the epidemiology and control of livestock and wildlife schistosomiasis. There are three main schistosome species occurring in African livestock, all of which are intestinal: *Schistosoma bovis*, *Schistosoma curassoni* and *Schistosoma mattheei*. Additionally, *Schistosoma margrebowiei* and *Schistosoma leiperi* are present in African bovine species, but livestock are thought to be secondary definitive hosts, i.e. they alone are not able to maintain transmission cycles [7]. Available data indicate that *S. bovis* occurs throughout Africa, but mostly north of 10° South [8]. South of this latitude, *S. mattheei* is the predominant species, while *S. curassoni* is located sympatrically with *S. bovis* in some parts of West Africa [8–10]. In 2018, the average prevalence of schistosomiasis across Côte d’Ivoire in slaughtered cattle, sheep and goats was estimated to be 22.5%, 1.3% and 1.2%, respectively [11].

Epidemiological investigations into livestock schistosomiasis are not commonly carried out; when they are, they rarely include molecular species identification because of limited resources. Usually, the infecting species are not known or are merely presumed based on egg morphology or the animal’s geographic origin. However, this can be imprecise, especially in the presence of *Schistosoma* hybrids. Natural hybridization within and between human and animal schistosomes has been suspected since the 1940s, and recently hybrids between a variety of *Schistosoma* species have been reported in humans, snails and wildlife in Benin, Cameroon, Corsica, Côte d’Ivoire, Kenya, Mali, Niger, Nigeria, Senegal, South Africa, Tanzania and Zambia [10, 12–21]. Few studies have investigated the occurrence of hybrids in livestock. Of the four studies published from Benin, Cameroon and Senegal, there has only been one tentative report of hybrids in livestock in Benin (*S. bovis* × *S. haematobium*). These hybrids were identified through cercarial emergence patterns and molecular markers (*ITS1/2*), although the data are not unequivocal and need further confirmation [13, 17, 18, 22]. Hybrids are concerning for a number of reasons. First, hybrids may reside at anatomical sites not normally occupied by schistosomes in those hosts (e.g. in veins of the urinary plexus in cattle), which may lead to false-negative diagnoses as those sites are not routinely examined [22–24]. Second, hybridization affects egg morphology, rendering species identification challenging [18, 25, 26]. Third, some hybrids have demonstrated hybrid vigour based on newly acquired advantageous traits such as increased virulence or transmission and host range expansion [10].

The poor understanding of the epidemiology of livestock schistosomiasis and the role of zoonotic interactions, in general, in the human schistosomiasis transmission cycle is of concern. Zoonotic reservoirs are an important component of the transmission cycle as they can harbour parasites and perpetuate transmission, threatening control and elimination efforts made by human schistosomiasis control programmes [27, 28]. Furthermore, zoonotic reservoirs can influence parasite evolution by providing opportunities for host switching or genetic exchange, which can lead to novel genetic combinations [28].

Genetic diversity, differentiation and structuring can reveal transmission dynamics and patterns that could impact the spread of advantageous gene variants and improve our understanding of the biology and options for preventive interventions. While genetic diversity and structuring have been investigated in *S. mansoni* and *S. haematobium* [29, 30], to date, we are aware of only two investigations into *S. bovis* population genetics in Africa [13, 22] and none in *S. curassoni*.

To better understand the role of livestock in schistosomiasis epidemiology, we identified schistosome species and clarified basic population genetic patterns of *Schistosoma* species from livestock in Côte d’Ivoire. This study is part of a larger project investigating transmission dynamics of schistosomes from a One Health perspective to deepen the understanding of the epidemiology and transmission cycles of these parasites in both human and animal hosts and produce new knowledge from a veterinary perspective.

## Methods

### Study area and sites

The study was carried out in Côte d’Ivoire, a country with 27.5 million people in 2020 and an estimated 1.34 million cattle in 1988 [31, 32]. Côte d’Ivoire has three ecozones: the South and West parts of the country with abundant rainfall and forest, the northern region with savannah and less rainfall and the northwestern part also with savannah but more rainfall than the North [33].

**Fig. 1:**
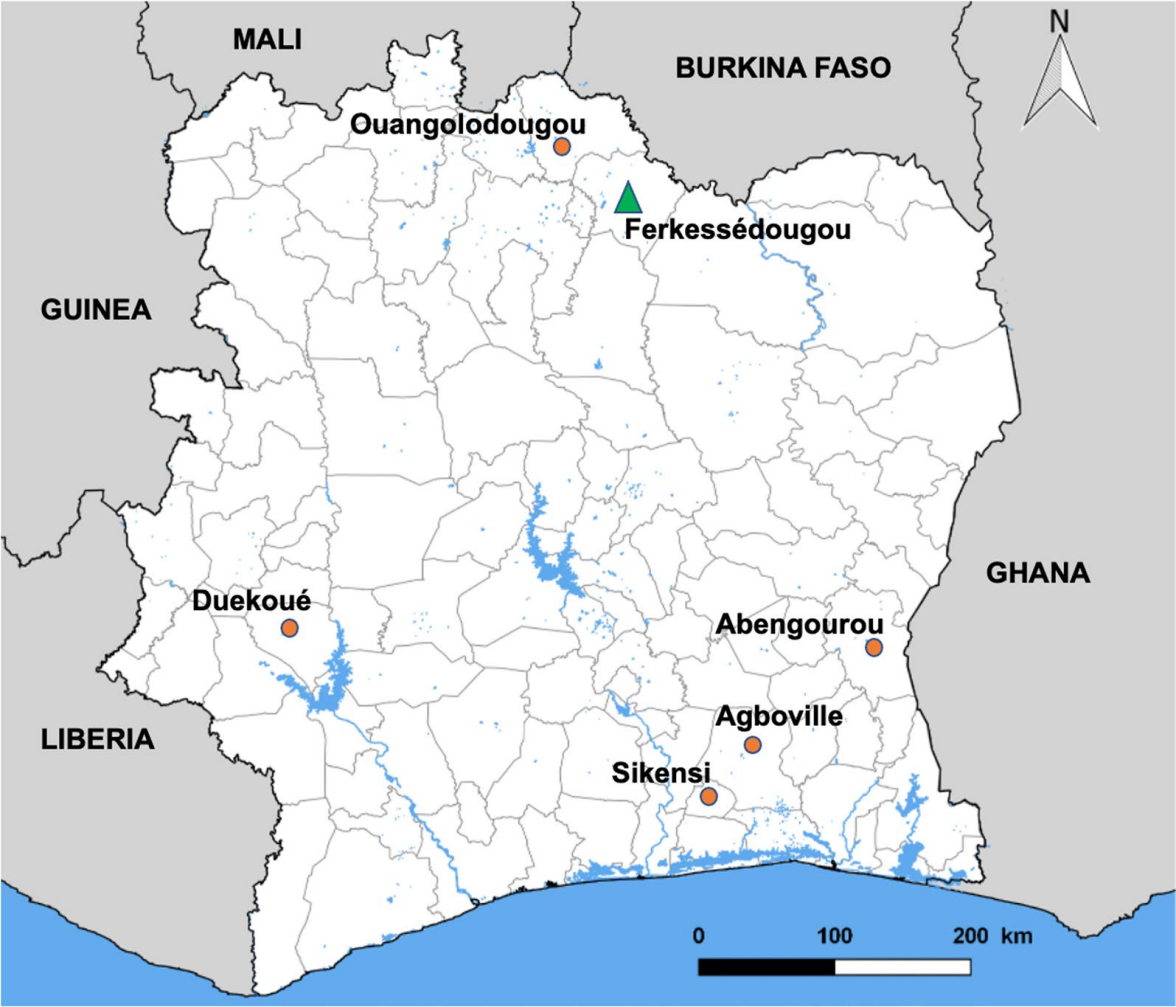
Map of Côte d’Ivoire. Orange circles indicate abattoir sites sampled for adult schistosomes from slaughtered cattle. In Ferkessédougou (green triangle), both slaughtered cattle from abattoirs and live cattle on farms were sampled for adult schistosomes and miracidia, respectively

The study involved sampling adult schistosomes from cattle, sheep and goats at six sites across Côte d’Ivoire in August 2018 and July 2019 (Fig. 1). Three sites were in the southeastern region of the country, one site in the west and two sites in the north-central region. Adult schistosomes were collected from slaughtered livestock in abattoirs at all six sites, while miracidia were sampled from live livestock on farms only in Ferkessédougou (north-central region). This sampling strategy was undertaken to compare population genetic patterns revealed by sampling different host populations and parasite life stages. As described in previous papers [11, 34], these sites were chosen because of the occurrence of human and animal schistosomiasis as well as the presence of small dams and water bodies frequented by both humans and livestock. As northern Côte d’Ivoire is the main livestock production area in the country, it is best suited for exploring the interactions between parasites and their host species, including humans, livestock and snails. Furthermore, in this region of Côte d’Ivoire, it is thought that elimination of schistosomiasis is feasible; hence, it is important to understand the transmission dynamics amongst all hosts [35].

### Specimen collection and storage

At each abattoir site, the mesenteric veins of cattle, sheep and goats were examined for adult schistosomes, as described in a previous publication [11]. If present, schistosomes were excised from the mesenteric veins and rinsed in phosphate-buffered saline (PBS); male and female schistosomes were separated and stored individually in 95% ethanol or in 190 µl lysis solution (0.1 M Tris-HCl, 1% sodium dodecyl sulphate) and 10 µl of (20 µg/µl) proteinase K at room temperature before being shipped to Switzerland. After the schistosomes in lysis solution appeared completely lysed (approximately 2–3 days), 200 µl of TE buffer (10 mM Tris and 1 mM EDTA) was added.

On farms in Ferkessédougou, faeces were manually extracted from the rectums of cattle, sheep and goats. Samples were analysed at the “Ministère des Ressources Animales et Halieutiques” (MIRAH) premises in Ferkessédougou by technicians from the parasitology departments of the three “Laboratoire National d’Appui au Développement Agricole” (LANADA) laboratories in charge of animal health. A walnut-size portion of faeces from each animal was washed, filtered and scraped with a wooden spatula through a drum filter (400-μm mesh size) with locally purchased bottled water. The filtrate was further filtered through a Pitchford filter [34]. The inner sieve mesh of the Pitchford filter was 300 μm and the outer sieve mesh was 30 μm, as described elsewhere [36]. The filtrate was drained into a Petri dish, which was left in a semi-shady area for 1 h to facilitate *Schistosoma* egg hatching. The Petri dishes were examined under 10 × magnification for miracidia and again every hour for 4 h. Miracidia were individually collected with a pipette in 5 μl of water, deposited on a Whatman Indicating Flinders Technology Associates (FTA) card (GE Healthcare Life Sciences; Buckinghamshire, UK) and left to dry for 1 h. Additionally, at the Ferkessédougou abattoir, five bladders were collected from the carcasses of cattle. As *S. haematobium* resides in the veins of the urinary plexus in humans, *S. haematobium* or *S. haematobium* hybrids might be excreted via urine in livestock. The urogenital veins were inspected for schistosomes and each urine was filtered through a 30-µm filter with locally purchased bottled water into a Petri dish. The Petri dishes were processed as described above for the collection of miracidia.

### DNA extraction

Molecular analyses were performed at the Swiss Tropical and Public Health Institute (Swiss TPH) in Basel, Switzerland. The schistosomes stored in 95% ethanol were rinsed in PBS and then deposited in 190 µl of lysis solution with 10 µl of (20 µg/µl) proteinase K, as described above, and incubated at 56 °C with agitation for 1 h. DNeasy blood and tissue column kits (Qiagen; Hilden, Germany) were used for DNA extraction of all schistosome samples. The manufacturer’s protocol was followed, except for the lysis solution used.

DNA from each miracidium was extracted using a Chelex® protocol (Bio-Rad Laboratories; Cressier, Switzerland). A 3-mm Harris Uni-core micro-punch (Qiagen; Hilden, Germany) was used to cut out the indicated spot where the miracidium was deposited on the FTA card. The punch was placed in a 1.5-ml Eppendorf tube with 100 µl of ultra-pure water and left to sit at room temperature for 10 min. The water was then removed with a pipette and the punch rinsed once more. Subsequently, 80 µl of 5% Chelex solution was added (Chelex solution was mixed on a magnetic agitator to allow distribution of Chelex beads) and incubated at 65 °C with agitation for at least 30 min. The temperature was increased to 99 °C for a final 8 min, after which the solution was centrifuged at 13,000 rotations per min (rpm) for 2 min. Finally, 60 µl of the DNA-containing supernatant was pipetted into a new Eppendorf with 5 µl of Low TE buffer (10 mM Tris and 0.1 mM EDTA) and stored at − 20 °C. Each extraction was performed with a negative control (double-distilled water instead of DNA) and negative controls were checked by PCR to ensure that samples were not cross-contaminated.

### Species identification using *cox1* and *ITS1/2* PCR

The *cox1* genetic profile of all 514 samples (400 adult schistosomes and 114 miracidia) was determined by amplicon size using a multiplex *cox1* PCR (*S. haematobium* 120 base pairs (bp); *S. mansoni* 215 bp; *S. bovis* and *S. curassoni* 260 bp) [16, 37]. The partial *cox1* from each sample was amplified in a 10-µl PCR containing 0.5 µl DNA, 5.0 µl Qiagen Multiplex PCR Master Mix (HotstarTaq DNA Polymerase, optimized salt concentrations, dNTPs and a unique PCR buffer) (Qiagen; Hilden, Germany), 3.5 µl distilled water, 1 µl primer mix (4 µM of each primer); three forward primers, Sh.F 5’-GGTCTCGTGTATGAGATCCTATAGTTTG-3’, Sm.F 5’-CTTTGATTCGTTAACTGGAGTG-3’ and Sb.F 5’-GTTTAGGTAGTGTAGTTTGGGCTCAC-3’, and one reverse primer, Shmb.R 5’-CAAGTATCATGAAAYARTATRTCTAA-3’ [38]. Positive (*S. bovis* from Côte d’Ivoire) and negative controls were run in every PCR. The PCR was initiated by 15 min at 95 °C, followed by 45 cycles of 94 °C for 10 s, 52 °C for 30 s and 72 °C for 10 s and a final extension of 72 °C for 2 min.

The PCR products and a PCR DNA ladder were loaded onto 2% agarose gel (Promega; Madison, WI, USA) with 0.5× Tris–borate-EDTA (TBE) (Thermo Scientific; Waltham, MA, USA) and run for 25 min at 75 V. Agarose gels were soaked in GelRed (Merck KGaA; Darmstadt, Germany) for at least 30 min before being visualized. Bands of 120 bp (*S. haematobium*), 215 bp (*S. mansoni*) and 260 bp (*S. bovis* and *S. curassoni*) were examined.

Due to limited resources, a random selection of the samples was chosen for PCR and sequencing of the *ITS1/2* locus to confirm species identification. The *ITS1/2* locus was amplified in a total volume of 10 µl consisting of 1 µl BD 10× buffer (Solis BioDyne; Tartu, Estonia), 1.3 µl 25 mM MgCl_2_, 0.8 µl 10 mM dNTP mix (2.5 mM of each dNTP), 0.1 µl 5U/ml FirePol® Taq polymerase (Solis BioDyne; Tartu, Estonia), 0.5 µl DNA, 5.3 µl water and 0.5 µl of each 10 µM forward (ITS5: 5’-GGAAGTAAAAGTCGTAACAAG-3’) and reverse primers (ITS4: 5’-TCCTCCGCTTATTGATATGC-3’). Each PCR was run with a positive (*S. bovis* from Côte d’Ivoire) and negative control. The PCR protocol was initiated with 4 min at 94 °C, followed by 40 cycles of 30 s at 94 °C, 30 s at 54 °C and 30 s at 72 °C and a final extension of 2 min at 72 °C. PCR products were sent to Microsynth (Balgach, Switzerland) for uni-directional Sanger sequencing using the reverse primer.

*ITS1/2* sequences were queried in GenBank using the Basic Local Alignment Search Tool (BLAST) and then aligned, cleaned and analysed in CodonCode Aligner version 6.0.2 (CodonCode Corp.; Centerville, MA, USA) with sequences of *S. haematobium* from collaborators as well as *S. curassoni* and *S. bovis* from GenBank (MT580947, MT580961.1 and FJ588862). Five species-specific single nucleotide polymorphisms (SNPs) in the *ITS1/2* locus that distinguish *S. haematobium* from *S. bovis*, one of which also distinguishes *S. bovis* from *S. curassoni*, were analysed for all samples [24, 39]. *ITS1/2* trace files were inspected for double chromatogram peaks to determine heterozygosity at the five SNPs [24, 30].

### Microsatellite analysis

A sub-selection of specimens from all sites (Table S1) were analysed using two panels of microsatellites markers [40]. Microsatellites were used as they have many alleles and a high mutation rate leading to high levels of informative variation that result in high resolution [41]. These microsatellite panels were developed for *S. haematobium*, a sister species of *S. bovis*, and therefore some loci produced poor results (C102, Shae_14, C131, Shae_07, Shae_10, Shae_08) and were removed from the analysis (Table 1) [13, 22]. Loci Shae_05 and Shae_15 were further omitted from analyses that included miracidia because they consistently produced poor results with miracidia DNA. Microsatellite analysis was also used for samples from Ferkessédougou to assess in greater detail how genetic diversity across the host population compares to that within individual hosts and to determine whether adult worms from slaughtered cattle yield the same patterns as miracidia collected from live animals. Each microsatellite panel was run as a multiplex PCR, as follows: 5 µl Qiagen Multiplex PCR Master Mix (Qiagen; Hilden, Germany), 1 µl DNA, 3 µl double-distilled water and 1 µl of 10 µM primer mix (0.8 µM of each primer in sterile water). Each PCR had two positive controls, *S. haematobium* and *S. bovis* (both originating from Côte d’Ivoire), as well as a negative control. The PCR protocol was initiated by 15 min at 95 °C, followed by 45 cycles of 94 °C for 30 s, 56 °C for 1.5 min and 72 °C for 1 min and a final extension of 72 °C for 5 min. PCR products were sent to Microsynth (Balgach, Switzerland) for fragment length analysis using the Applied Biosystems 3730XL DNA Analyzer with size standard GS500LIZ.

**Table 1:** *Schistosoma* microsatellite panels, loci used, allele size ranges (base pairs) and fluorescent dye. Loci Shae_05 and Shae_15 were omitted from analyses including miracidia

Panel	Locus	Allele range	Fluorescent dye
Panel 1	C111	185–209	ATTO565 (red)
	Shae_01	240–282	HEX (green)
	Shae_03	291–375	FAM (blue)
	Shae_06	305–331	ATTO550 (yellow)
	Shae_09	190–241	FAM (blue)
Panel 2	Shae_02	152–224	ATTO550 (yellow)
	Shae_04	264–313	FAM (blue)
	Shae_05	261–291	ATTO550 (yellow)
	Shae_11	172–209	HEX (green)
	Shae_12	241–262	ATTO565 (red)
	Shae_13	163–229	FAM (blue)
	Shae_15	270–297	HEX (green)

The trace files were imported into Genemapper (v. 6.0, ThermoFisher Scientific; Waltham, MA, USA), automatic bin calling was established based on the samples, and all automatically called peaks were confirmed at least twice by visual inspection. We experienced some difficulties distinguishing the fluorescent dyes as there was leakage between green, blue and yellow, which proved difficult for peak calling when allele ranges between colours overlapped. Samples which displayed questionable peaks were amplified and fragment analysed again in multiplex PCR to confirm the call.

### Genetic diversity measures and genotypic differentiation

Rarified allelic richness (*A*_*r*_) and fixation index (*F*_IS_) with 95% confidence intervals (CIs; bootstrapped with 999 repetitions) were calculated using the function *basicStats* from the package diveRsity, version 1.9.90 [42], in R version 4.0.2 [43]. Observed (*H*_*o*_) and expected heterozygosity (*H*_*e*_) with 95% CIs (bootstrapped with 1000 repetitions) as well as mean, minimum, median and maximum number of alleles (*A*) across loci were calculated using functions *popgen* and *bootstrapHet* from the package PopGenKit, version 1.0 [44], in R version 4.0.2 [43]. Probability of deviation from Hardy-Weinberg equilibrium (*P*_HWE_) was calculated in genepop version 4.7.5 [45] using option 1, suboption 3. For all other diversity measures, where not otherwise stated, statistical significance was determined by comparing the 95% CIs, deeming any overlap of CI arms of ≤ 50% as significant, as suggested by Cumming and colleagues [46]. Differentiation was measured by *F*_ST_ and *R*_ST_ calculated in RStudio with the package genepop version 1.1.7 [45]. Significance of genotypic differentiation was calculated in the same package using Fisher’s exact test for all population pairs.

### Population structure

Population structure analysis was completed in structure version 2.3.4 [47], using the automation implemented in strauto version 1 [48]. All analyses were run with a burn-in of 50,000 and 500,000 Markov chain Monte Carlo (MCMC) iterations after burn-in. Each *K* from 1 to 16 was run 10 times, and then the best *K* (best number of clusters or subpopulations) was determined by the Evanno method in structure harvester version 0.6.93 [49]. The results of the 10 runs at the best *K* were averaged in clumpp version 1.1.2 [50] and used to produce plots visualizing the contribution of each genetic cluster to the genome make-up of each individual parasite. Discriminant analysis of principal components (DAPC) was performed and results plotted in rstudio using the package adegenet version 2.1.3 [43, 51].

### Geographic population structure of adult schistosomes across Côte d’Ivoire

To compare genetic diversity and investigate geographic differentiation across Côte d’Ivoire, microsatellite data from a subsample of the 400 adult schistosomes were analysed. To enhance data independence, the number of hosts analysed per sampling site was maximised, but only one worm pair (i.e. paired male/female worm) per host individual.

### Genetic differences between male and female schistosomes

The sampling scheme allowed for a highly controlled comparison of the genetic diversity and population genetic patterns of male and female schistosomes across Côte d’Ivoire. For this analysis, schistosomes from all locations that were collected either as couples or (in few cases) as the only worm from one host were included and more than one couple per host individual were included whenever available.

### Comparison of within- and between-host genetic diversity of adult schistosomes

A subsample of the microsatellite data from the adult schistosomes in Ferkessédougou was subjected to within- and between-host genetic diversity. This was to compare the genetic diversity of adult schistosome populations in different hosts to determine whether analyses based on many parasites from a few hosts are representative of the population across host individuals. Within-host diversity was assessed in the “within group” consisting of schistosomes populations from three slaughtered cattle hosts, from which 19, 20 and 22 adult schistosomes were analysed. Most data were from schistosomes that were originally coupled males and females at collection.

Between-host diversity was assessed in the “between group”, consisting of 84 adult schistosomes from 39 cows: four singleton worms (one per host) from four slaughtered cattle, 33 couples (66 worms in total) from 33 slaughtered cattle (one couple per host) and another 14 worms from two slaughtered cattle (8 worms from one and 6 from the other).

For statistical analysis, 10 rounds of random subsampling were performed. In each subsampling round, two groups of 10 adult schistosomes were randomly drawn without replacement from each of the three “within” cows and four groups of 10 adult schistosomes were drawn from the “between group”. The following rules were applied to the subsampling: in the “within” cows, the schistosomes were randomly assigned to two groups; each group had a total of 10 schistosomes. In the cow with 22 schistosomes, two were randomly excluded at the start of any of the 10 rounds. In the cow with 19 schistosomes, one worm was randomly drawn twice. In the “between group”, in every round, the first four schistosomes were randomly excluded (3 from the cow with 8 worms, 1 from the cow with 6 worms). The remaining 80 adult schistosomes were randomly assigned to 8 groups of 10 worms each. In the end, this procedure resulted in 20 randomly drawn groups of 10 schistosomes for each of the three “within-cows” and in 80 randomly drawn groups of 10 worms for the “between group”. Diversity measures were calculated for each group of 10 schistosomes and differences between the “between group” and the three cows of the “within group” were assessed by a linear model using function *lm* in R version 4.0.2 [43].

### Genetic differentiation between adult schistosomes and miracidia in Ferkessédougou

Patterns of genetic differentiation between adult schistosomes and miracidia populations from Ferkessédougou were compared. Genetic indices and population structure of 30 miracidia obtained from 30 live cattle (one miracidium per host) were compared to 80 schistosomes obtained from 42 slaughtered cattle (one couple or one singleton per host). In a second analysis, the amount of genetic diversity occurring within hosts (many parasites per host), specifically the differences between within-host diversity of slaughtered cattle (adult schistosomes) and live cattle (miracidia), was investigated. For this analysis, data from at least 10 samples from each of eight host individuals (22, 20 and 19 adult schistosomes from three slaughtered cattle and 17, 16, 11, 10 and 10 miracidia from five live cattle) were used. The mean index values for each host individual were used to determine significant differences between the two approaches using linear models, i.e. function *lm* in R version 4.0.2 [43].

## Results

### Molecular *Schistosoma* species determination

A total of 400 adult schistosomes from 86 slaughtered cattle across the six sites (Fig. 1) and 114 miracidia from 30 live cattle in Ferkessédougou were collected and their DNA extracted (Additional file 1: Table S1). Adult schistosomes and miracidia from sheep and goats were not included in the analysis as the prevalence of schistosomiasis was low, and therefore the number of specimens collected from these hosts was too small to justify analysis. All the bladders and urine collected from slaughtered cattle in Ferkessédougou were negative for schistosomes or eggs.

Three hundred seventy-one adult schistosome (93%) and 101 miracidia (89%) produced *cox1* amplicons, all of which were 260 bp in length, indicating that all adult schistosomes and miracidia had mitochondrial DNA of *S. bovis* or *S. curassoni*.

Of the 107 adult schistosomes subjected to *ITS1/2* PCR, 101 (94%) produced data (GenBank accession numbers: PP312934-3034). All samples showed an *ITS1/2* SNP profile identical to *S. bovis*. Of the 34 miracidia from cattle that were subjected to the same PCR, only four produced sequences and they too were consistent with the SNP profile for *S. bovis* (GenBank accession numbers: PP313051-54).

### Geographic population structure of adult schistosomes across Côte d’Ivoire

The geographic analysis explored genetic diversity at different locations and structuring between the locations. A total of 146 adult schistosomes from 76 hosts across the six sites were included in the analysis. All sites showed high levels of genetic variability as measured by *A*_*r*_, *H*_*o*_ and *H*_*e*_. Values were similar across all sites, except for Abengourou (Table 2). *H*_*e*_ exhibited highly skewed CIs because it approached or reached 1.0 in several loci at all sites. Significant deviation from HWE and high levels of inbreeding were apparent at all six sites, but with little variation between sites.

**Table 2:** Genetic diversity indices for schistosomes from cattle at six locations across Côte d’Ivoire based on 12 microsatellite loci

Location	*N*	*N*_*H*_	*A*	*A*_*r*_	*H*_*o*_	*H*_*e*_	*P*_HWE_	*F*_IS_
Ouangolodougou	21	11	9.92	4.78	0.556	0.754	2.7 × 10^–12^	0.238
			(3, 18)	(3.75–5.58)	(0.484–0.619)	(0.679–0.773)		(0.129–0.308)
Ferkessédougou	80	42	11.25	5.00	0.574	0.725	1.2 × 10^–15^	0.222
			(4, 22)	(4.33–5.58)	(0.543–0.598)	(0.703–0.73)		(0.178–0.255)
Abengourou	6	3	4.92	3.88	0.528	0.644	1.9 × 10^–2^	0.185
			(2, 11)	(3.00–4.58)	(0.444–0.611)	(0.468–0.650)		(-0.042–0.224)
Duekoué	14	7	7.25	4.46	0.527	0.679	1.1 × 10^–8^	0.248
			(2, 14)	(3.33–5.42)	(0.468–0.577)	(0.599–0.687)		(0.135–0.292)
Agboville	14	7	8.50	4.97	0.600	0.744	2.1 × 10^–7^	0.199
			(3, 14)	(3.67–5.92)	(0.542–0.652)	(0.671–0.737)		(0.055–0.248)
Sikensi	11	6	6.00	4.15	0.542	0.674	1.8 × 10^–6^	0.24
			(2, 11)	(3.33–4.83)	(0.472–0.601)	(0.578–0.677)		(0.054–0.295)

*N*, number of schistosome samples (maximally two per host individual); *N*_*H*_, number of host individuals from which schistosomes were collected; *A*, average (minimum, maximum) number of alleles across loci; *A*_*r*_, mean (+ 95% CI) allelic richness across loci; *H*_*o*_, mean (+ 95% CI) observed heterozygosity across loci; *H*_*e*_, mean (+ 95% CI) estimated heterozygosity across loci; *P*_HWE_, probability of deviation from Hardy-Weinberg equilibrium; *F*_IS_, mean (+ 95% CI) fixation index across loci

**Fig. 2:**
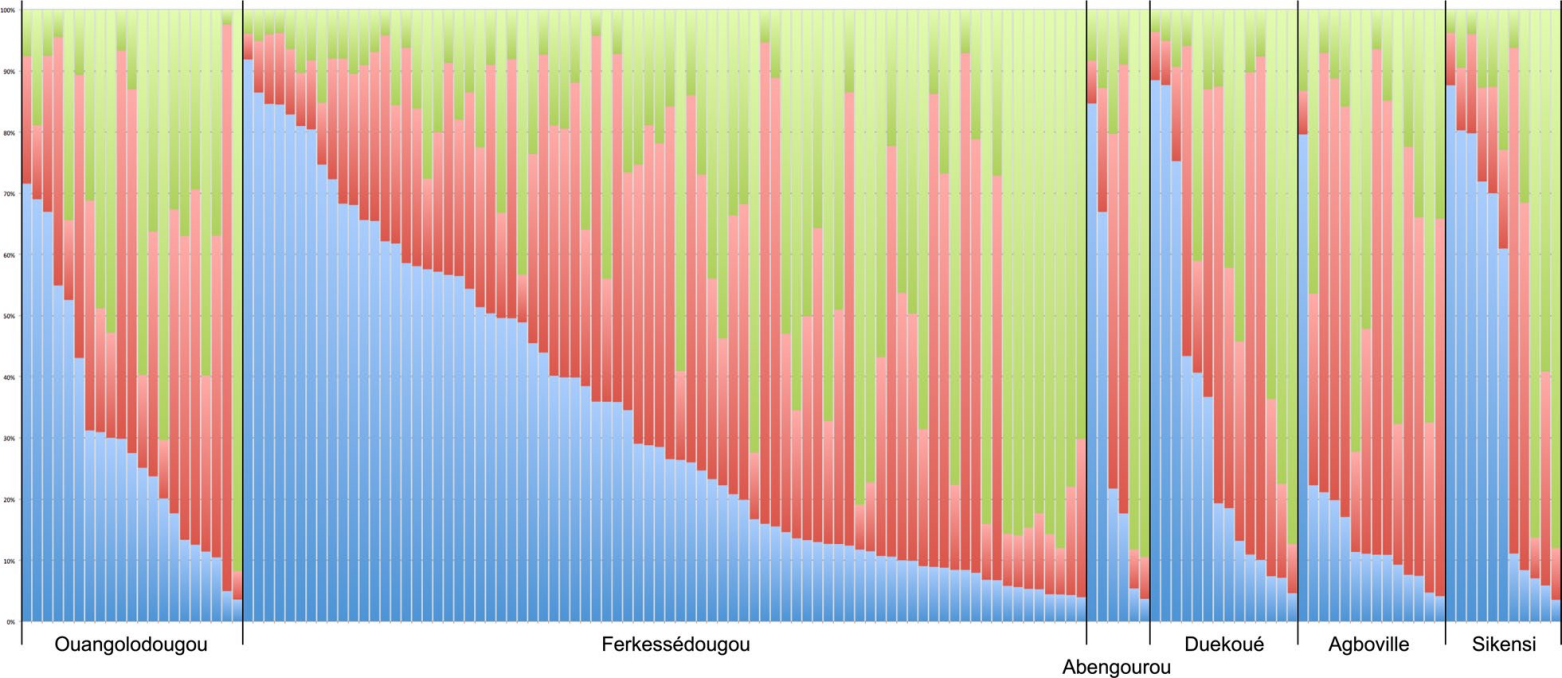
Plot of structure analysis of schistosomes from cattle across Côte d‘Ivoire. Each column represents one schistosome with the colours indicating the relative contribution of the three genetic clusters (*K* = 3) to the parasite’s genome

**Fig. 3:**
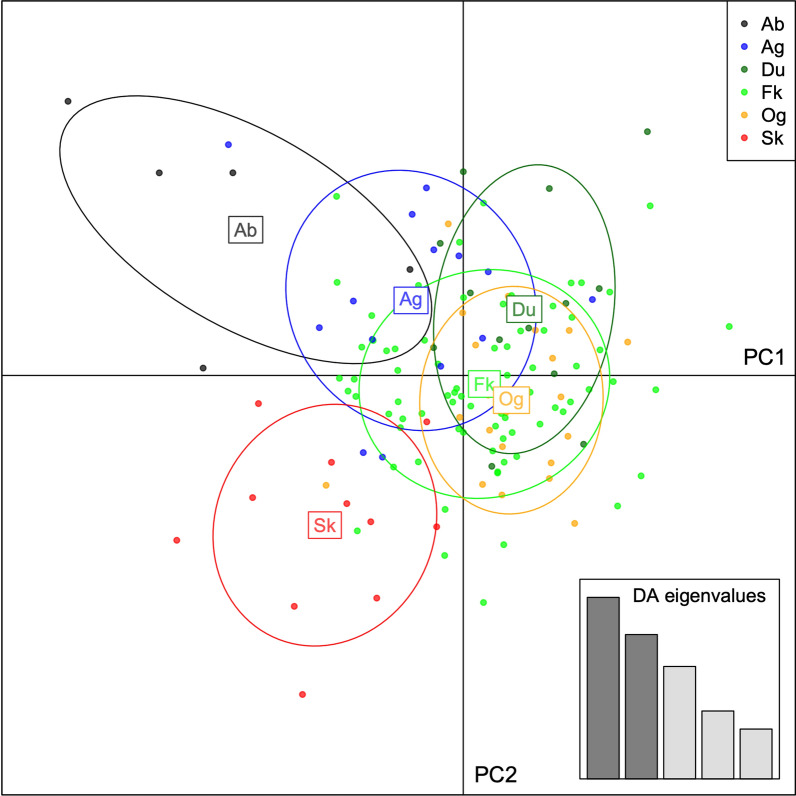
Scatterplot of the first two components of discriminant analysis of principal components (DAPC) of populations of schistosomes from cattle, based on geography. Dots are individuals, circles inertia ellipses. Forty principal components were retained, representing 84.3% of the total variation in allele frequencies. Ab, Abengourou; Ag, Agboville; Du, Duekoué; Fk, Ferkessédougou; Og, Ouangolodougou; Sk, Sikensi

All *F*_ST_ and *R*_ST_ values between geographic locations were close to zero (highest *F*_ST_, 0.022; highest *R*_ST_, 0.055), indicating no or very little differentiation between populations (values not shown). All values but one were non-significant (*P*-values > 0.15), the exception being Duekoué vs. Sikensi (*F*_ST_ = 0.008, *P* = 0.045). This F_ST_ value was close to zero, indicating no differentiation. Likewise, structure analysis revealed no structuring among the six locations (Fig. 2). Evanno’s method suggested three genetic clusters (*K* = 3) (Additional file 1: Fig. S1A) with similar relative representation in the parasites’ genomes in all six sites. The only sites that showed some level of genetic differentiation were Agboville and Sikensi (Fig. 2). DAPC analysis indicated little differentiation between locations with Sikensi and Abengourou being most distinct (Fig. 3).

### Genetic differences between male and female schistosomes

To determine whether there are genetic differences between male and female schistosomes, 295 adult schistosomes, mostly from couples, were analysed. *A*_*r*_ and *H*_*e*_ were comparable in both sexes, but the sexes significantly differed in *H*_*o*_. There were significant deviations from HWE and high levels of inbreeding in both sexes, with higher levels in females than males (Table 3).

**Table 3:** Genetic diversity indices of male and female schistosomes obtained from slaughtered cattle across Côte d’Ivoire based on 12 microsatellite loci

Fluke sex	*N*	*A*	*A*_*r*_	*H*_*o*_	*H*_*e*_	*P*_HWE_	*F*_IS_
Female	134	12.3	11.46	0.536	0.725	2.3 × 10^–15^	0.270
		(5, 23)	(11.00 -11.83)	(0.516–0.552)	(0.707–0.729)		(0.237–0.298)
Male	161	12.8	11.77	0.594	0.736	3.2 × 10^–20^	0.222
		(6, 25)	(11.25 -12.25)	(0.571–0.608)	(0.721–0.739)		(0.190–0.247)

*N*, number of schistosome samples; *A*, average (minimum, maximum) number of alleles across all loci; *A*_*r*_, mean (+ 95% CI) allelic richness across all loci; *H*_*o*_, mean (+ 95% CI) observed heterozygosity across all loci; *H*_*e*_, mean (+ 95% CI) estimated heterozygosity across all loci; *P*_HWE_, probability of deviation from Hardy-Weinberg equilibrium; *F*_IS_, mean (+ 95% CI) fixation index across all loci

**Fig. 4:**
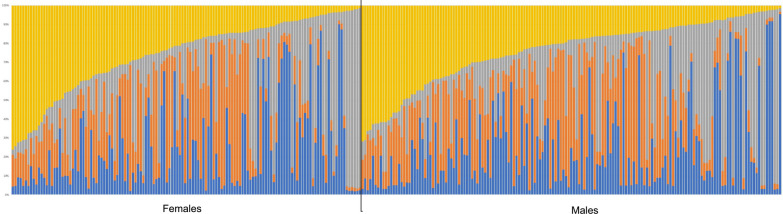
STRUCTURE analysis of male and female schistosomes obtained from slaughtered cattle across Côte d’Ivoire (*K* = 4) (see Additional file 1: Fig. S4)

There appeared to be no genotypic differentiation between males and females, with *F*_ST_ and *R*_ST_ values of 0.002 and 0.015, respectively. This was confirmed by the structure analysis, where Evanno’s method suggested four genetic clusters (Additional file 1: Fig. S1B), but no population structuring was evident between the two sexes (Fig. 4).

### Comparison of within- and between-host genetic diversity in adult schistosomes

**Fig. 5:**
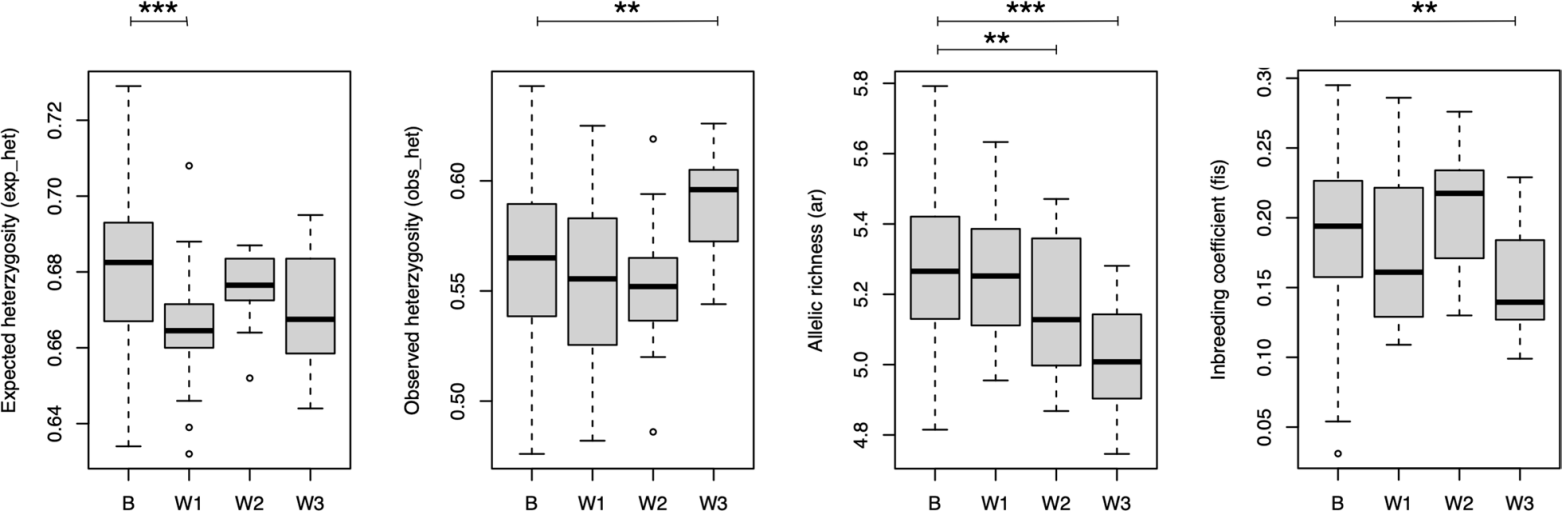
Boxplots of diversity measures from randomly drawn schistosomes across all cattle hosts (between, “B”) and within three individual cattle hosts (within, “W1”, “W2”, “W3”) in Ferkessédougou. **Significant pairwise difference at *P* < 0.01; ***significant pairwise difference at *P* < 0.001. See Additional file 1: Table S2 for detailed statistical values

The comparison of diversity measures within individual host animals to that across all host animals revealed statistically significant but moderate differences (Fig. 5, Additional file 1: Table S2). Genetic diversity within the three cow individuals, for which 19, 20 and 22 adult schistosomes were analysed, was lower than across the entire population for *H*_*e*_, *A*_*r*_ and *F*_IS_, while *H*_*o*_ was the same or even higher. However, in all measures, the differences were slight and for every measure one or two of the assessed cows were indistinguishable from the overall population.

### Genetic differentiation between adult schistosomes and miracidia in Ferkessédougou

**Fig. 6:**
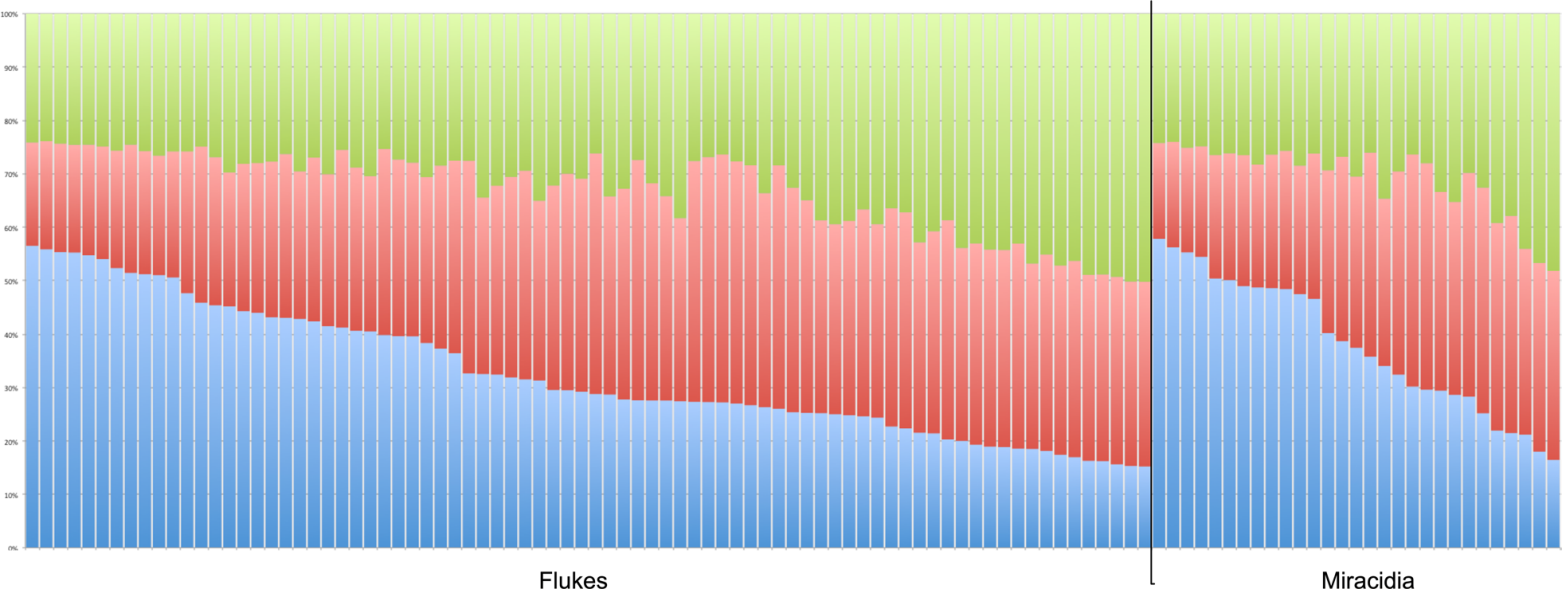
Structure analysis of miracidia from live cattle and adult schistosomes from slaughtered cattle in Ferkessédougou, Côte d’Ivoire, in 2018 and 2019 (*K* = 3)

All diversity values except *F*_IS_ were lower in miracidia than adult schistosomes, but differences were statistically significant only in *H*_*o*_ and *F*_IS_ (Table 4), which was strikingly higher in miracidia than in adult schistosomes and in any other analysis in this study. Deviation from HWE was apparent in both populations. Low genotypic differentiation was apparent between the populations (*F*_ST_ = 0.011 and *R*_ST_ = 0.025). Likewise, structure analysis showed no structuring between the two life stages. The analysis of Evanno’s K suggested that the genetic background of the schistosomes investigated was best explained by three genetic clusters (Additional file 1: Fig. S1C). The assignments of each parasite’s genome to these clusters were not markedly different between the life stages (Fig. 6).

**Table 4:** Genetic diversity indices for schistosomes from live farm cattle and slaughtered cattle from abattoirs in Ferkessédougou, Côte d’Ivoire, based on 10 microsatellite loci

Life stage	*N*	*N*_*H*_	*A*	*A*_*r*_	*H*_*o*_	*H*_*e*_	*P*_HWE_	*F*_IS_
Flukes	80	42	12.7	9.83	0.652	0.787	5.4 × 10^–13^	0.188
			(5, 22)	(9.10–10.60)	(0.620–0.676)	(0.766–0.790)		(0.140–0.221)
Miracidia	29	29	9.9	8.50	0.509	0.772	1.6 × 10^–22^	0.379
			(3, 15)	(7.60–9.20)	(0.444–0.536)	(0.701–0.770)		(0.302–0.425)

*N*, number of schistosome samples (maximally two per host individual); *N*_*H*_, number of host individuals from which schistosomes were collected; *A*, average (minimum, maximum) number of alleles across loci; *A*_*r*_, mean (+ 95% CI) allelic richness across loci; *H*_*o*_, mean (+ 95% CI) observed heterozygosity across loci; *H*_*e*_, mean (+ 95% CI) estimated heterozygosity across loci; *P*_HWE_, probability of deviation from Hardy-Weinberg equilibrium (stars highlight significance at < 0.05); *F*_IS_, mean (+ 95% CI) fixation index across loci

Within-host genetic diversity between adult schistosomes and miracidia showed a deficiency of heterozygotes and statistically significant deviation from HWE in all hosts (Table 5). Reiterating the result of the first analysis (Table 4), *A*_*r*_ and *H*_*e*_ were generally higher in adult schistosomes than miracidia and *F*_IS_ was higher in miracidia in every host individual. When taking the mean values per host individual as entries for linear models, the models showed significant differences between adult schistosomes and miracidia in A_r_ (*F* = 7.9, *P* = 0.031), *H*_*o*_ (*F* = 15.3, *P* = 0.008) and *F*_IS_ (*F* = 8.8, *P* = 0.025), but not *H*_*e*_ (*F* = 2.9, *P* = 0.138). Average number of alleles across loci (*A*) was not assessed because it is sensitive to sample size.

**Table 5:** Genetic diversity indices for schistosomes from within individual cattle hosts based on 10 microsatellite loci

Life stage	Host	*N*	*A*	*A*_*r*_	*H*_*o*_	*H*_*e*_	*P*_HWE_	*F*_IS_
Flukes	Cfr20	20	9.7	6.54	0.628	0.753	2.5 × 10^–6^	0.181
			(3, 17)	(5.50–7.40)	(0.567–0.685)	(0.699–0.758)		(0.058–0.256)
Flukes	Cfr78	18	8.8	6.30	0.627	0.75	1.3 × 10^–6^	0.177
			(3, 15)	(5.20–7.40)	(0.558–0.687)	(0.681–0.757)		(0.059–0.236)
Flukes	Cfr84	22	8.7	6.13	0.664	0.751	1.1 × 10^–7^	0.139
			(3, 14)	(5.10–7.00)	(0.617–0.707)	(0.704–0.755)		(0.043–0.189)
Miracidia	195	17	7.6	5.70	0.585	0.741	2.8 × 10^–7^	0.229
			(2, 13)	(4.70–6.60)	(0.494–0.654)	(0.666–0.732)		(0.072–0.333)
Miracidia	232	10	5.8	4.75	0.490	0.642	3.1 × 10^–5^	0.239
			(3, 10)	(3.80–5.50)	(0.455–0.525)	(0.531–0.650)		(0.063–0.282)
Miracidia	236	11	6.3	5.07	0.560	0.677	2.2 × 10^–4^	0.235
			(2, 12)	(4.20–5.80)	(0.486–0.625)	(0.566–0.693)		(0.087–0.269)
Miracidia	BV34	16	8.3	6.08	0.523	0.765	5.2 × 10^–15^	0.363
			(3, 14)	(5.10–6.91)	(0.460–0.564)	(0.670–0.763)		(0.254–0.405)
Miracidia	BV04	10	5.6	4.61	0.475	0.643	2.3 × 10^–7^	0.325
			(2, 8)	(3.60–5.40)	(0.370–0.570)	(0.517–0.657)		(0.011–0.444)

Schistosomes from slaughtered cattle Cfr20, Cfr78 and Cfr84 and miracidia from live cattle 195, 232, 236, BV34 and BV04 on farms in Ferkessédougou, Côte d’Ivoire

Host, host ID; *N*, number of parasite samples; A, average (minimum, maximum) number of alleles across loci; *A*_*r*_, mean (+ 95% CI) allelic richness across loci; *H*_*o*_, mean (+ 95% CI) observed heterozygosity across loci; *H*_*e*_, mean (+ 95% CI) estimated heterozygosity across loci; P_HWE_, probability of deviation from Hardy-Weinberg equilibrium; *F*_IS_, mean (+ 95% CI) fixation index across loci

Genotypic differentiation between the life stages as measured by *F*_ST_ and *R*_ST_ was weak with the highest *F*_ST_ and *R*_ST_ values being 0.048 and 0.055, respectively (Additional file 1: Table S3). Values between life stages were not significantly different from those within life stages (linear models, *F* = 1.06, *P* = 0.31 and *F* = 0.75, *P* = 0.39 for *F*_ST_ and *R*_ST_, respectively).

**Fig. 7:**
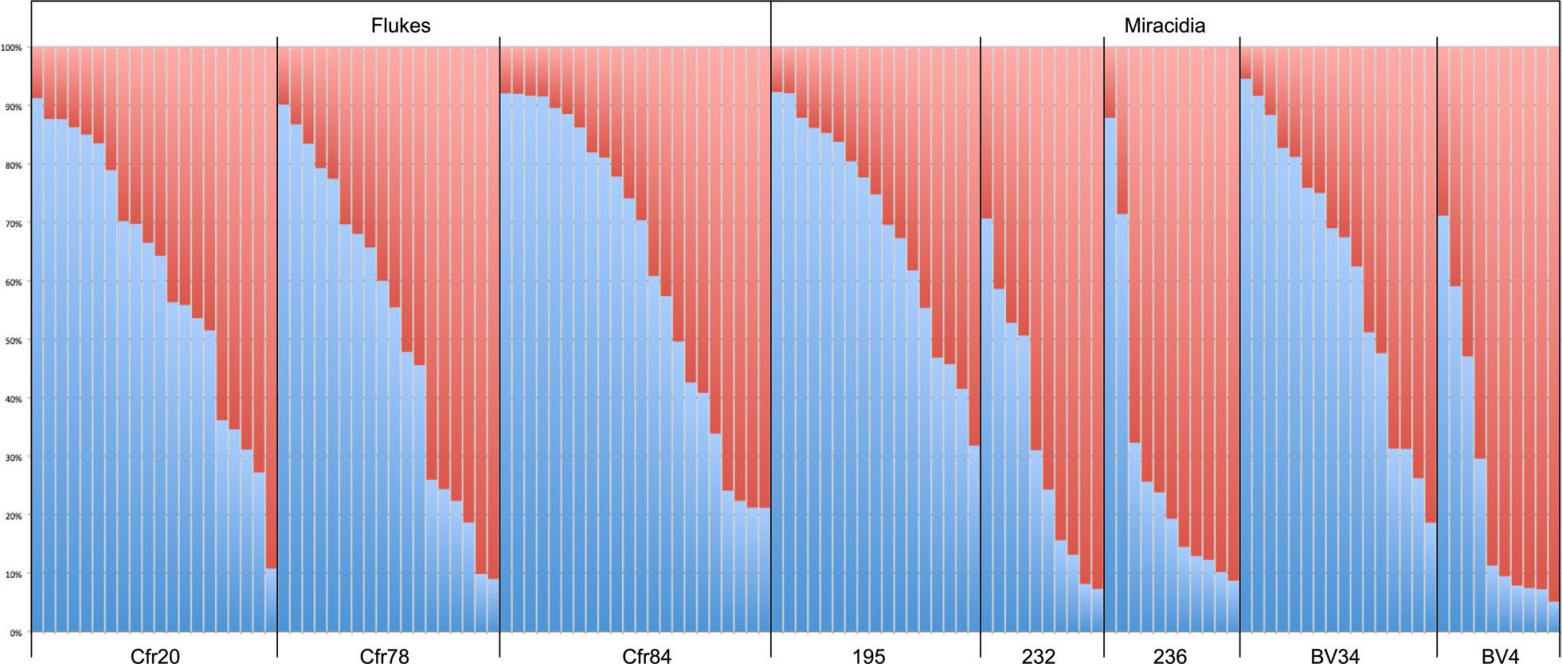
Structure analysis of miracidia and adult schistosomes from within live and slaughtered cattle, respectively, in Ferkessédougou, Côte d’Ivoire, in 2018 and 2019 (*K* = 2). Individual codes are as in Table 5

**Fig. 8:**
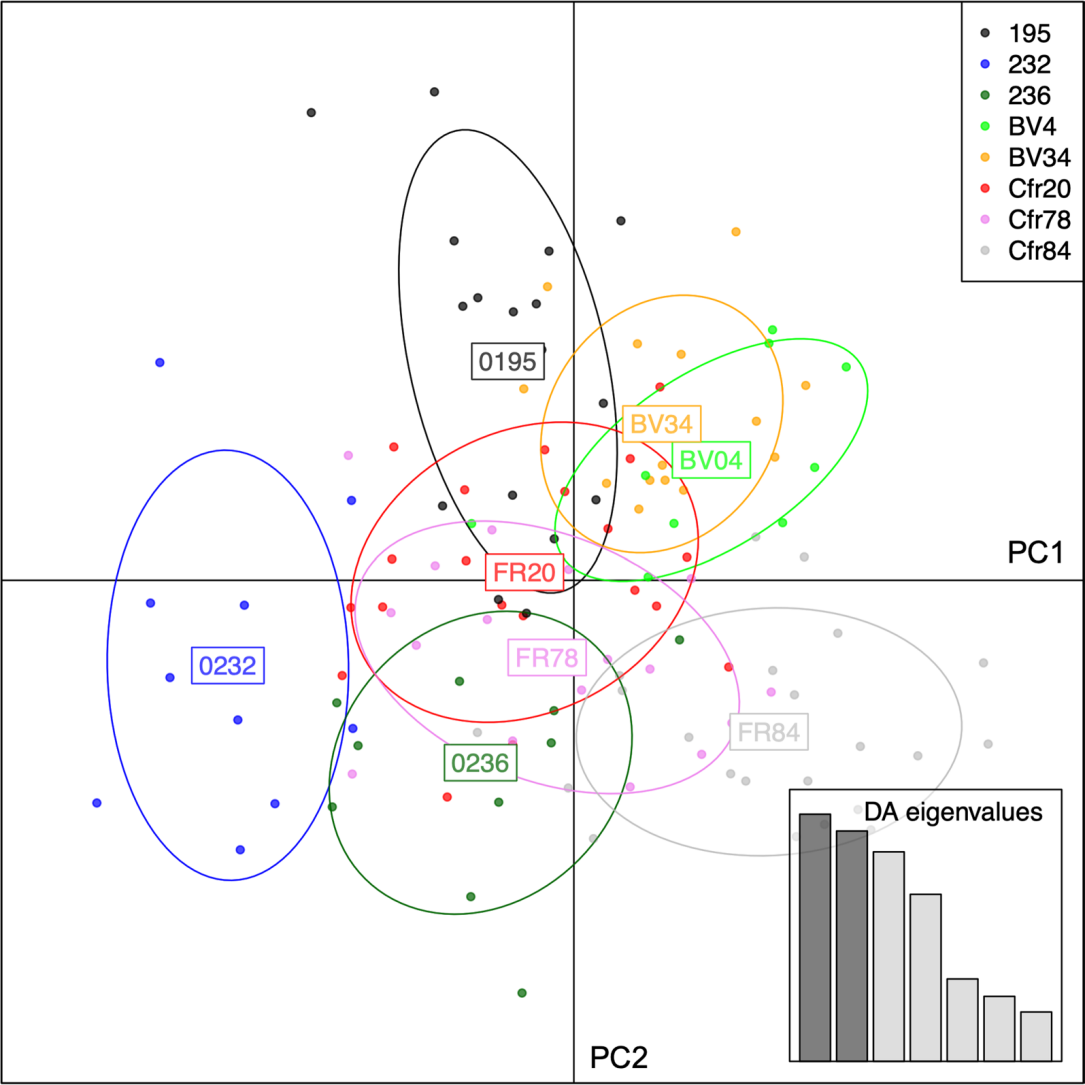
Scatterplot of the first two components of discriminant analysis of principal components (DAPC) of schistosomes from within slaughtered and live cattle hosts. Dots are individuals, circles inertia ellipses. Forty principal components were retained, representing 86.7% of the total variation in allele frequencies. Individual codes are as in Table 5

Likewise, structure analysis revealed two genetic clusters (Additional file 1: Fig. S1D), but no consistent differentiation between adult schistosomes and miracidia (Fig. 7). Three of the five miracidia populations from live cattle (232, 236 and BV4) appeared to be different from adult schistosomes in slaughtered cattle but the other two live cattle populations were indistinguishable. DAPC showed little differentiation between populations and, in particular, no differentiation between adult schistosomes and miracidia populations (Fig. 8).

## Discussion

To our knowledge only a few investigations have used molecular approaches to explore transmission dynamics and identify infecting *Schistosoma* species in African cattle, namely in Benin, Cameroon and Senegal [13, 17, 18, 22, 24]. All *cox1* and *ITS1/2* results from adult schistosomes and miracidia from cattle in Côte d’Ivoire exhibited typical *S. bovis* profiles. There were no indications of *S. haematobium*, *S. bovis* × *S. haematobium* hybrids (i.e. hybrids of livestock and human schistosomes) or *S. curassoni* in cattle at any of the six locations in Côte d’Ivoire. Our results thus mirror findings from Cameroon and Senegal, where cattle were also found to harbour only pure *S. bovis* [13, 17, 22]. To date, there has been only one tentative report of *S. bovis* × *S. haematobium* hybrids in cattle determined by cercarial shedding and molecular markers (*ITS1/2*) from eggs in stool samples in Benin [17]. However, the data are not unequivocal and hence need further confirmation. Hybrids of *S. bovis* × *S. haematobium* have, however, been reported in humans in Benin, Cameroon, Côte d’Ivoire, Niger, Nigeria and Senegal, as determined by *cox1* and *ITS1/2* loci [13, 20, 21, 24, 38, 39, 52, 53]. The studies from Côte d’Ivoire sampled the same sites as this study.

*Schistosoma haematobium* are normally found in the plexus of the urinary bladder in humans, as opposed to *S. bovis* and *S. curassoni*, where they are found in the mesenteric veins of cattle, respectively. In the current study, we examined five bladders and urine samples from slaughtered cattle, but we found neither adult schistosomes nor eggs. We attempted to collect urine samples from live cattle; however, these samples were difficult to obtain. Our findings are in line with those of Léger and colleagues [17] from Senegal, who, after examining vesical blood vessels and urine from 60 slaughtered cattle and 69 live cattle, found neither eggs nor miracidia, except in one cow. In this cow, *S. bovis* adult worms were found in the vesical blood vessels.

Our study revealed no indication of *S. curassoni*. However, it should be noted that distinguishing *S. curassoni* from *S. haematobium* and *S. bovis* using the *cox1* PCR and *ITS1/2* SNPs is not straightforward as neither method can definitively distinguish *S. curassoni* from *S. bovis*, *S. haematobium* and their hybrids [24]. *Schistosoma curassoni* has been reported in Mauritania [54], Mali, Senegal (enzymatic, morphological and molecular analysis) [17, 25] and Niger (egg morphology) [55] and hybrids of *S. bovis* × *S. curassoni* have been reported in livestock in Mali and Senegal [17, 25] and in a child in Niger [27]. The absence of *S. curassoni* in our samples from cattle may be due to the demonstrated lack of their preferred intermediate host snail, *Bulinus umbilicatus* [15, 56]. However, given their presence in livestock in West Africa [25, 27, 55] and the threat of livestock × human schistosome hybrids in general, improved molecular methods to distinguish *S. curassoni* are warranted. Since our investigation, one such tool, a tetra-primer amplification refractory mutation system PCR (T-ARMs- PCR), has been developed [57].

Along with data from Ivorian humans [38, 53], our results support the proposed multi-host, multi-parasite transmission cycle for the *S. haematobium* group of schistosomes in West Africa [17]. In this cycle, transmission of *S. haematobium* and *S. haematobium* × *S. bovis* hybrids occurs exclusively in humans with occasional spill-over of *S. bovis* from livestock. The barrier between human and animal transmission remains mostly intact but is occasionally punctured by animal parasites infecting humans. Evidence from Corsica [14] and Senegal [17] further supports this transmission cycle with the current data indicating that *S. bovis* can only complete its life cycle in humans if it pairs with *S. haematobium*. In Malawi, a similar model has been proposed for transmission cycles involving humans, cattle, *S. haematobium*, *S. mattheei* and *S. haematobium* × *S. mattheei* hybrids [23].

Schistosomes from cattle across the six sites in Côte d’Ivoire appear to be panmictic. There is generally an excess of homozygotes, high levels of inbreeding and little structuring or differentiation across all analyses performed (location, life stage, between hosts and by parasite sex). These observations are congruent with findings from cattle in Senegal and Cameroon [13, 22]. Compared to other *Schistosoma* species, it is acknowledged that *S. haematobium* is less genetically diverse than *S. mansoni* and *S. bovis* [13, 21, 22, 30, 38], whereas recent evidence suggests that *S. bovis* is less structured than *S. mansoni* and *S. haematobium* [13, 20, 21, 30, 38]. The decreased genetic diversity of *S. haematobium* is hypothesized to be the result of a recent and important bottleneck in the population, before recent expansion [30]. While large effective population size and the unrestricted movement of cattle across vast distances is thought to be the reason for less structuring in the *S. bovis* populations [30]. It appears that there are few barriers to gene flow of schistosomes in cattle, even across large distances, as found in this study, with > 500 km between Sikensi and Ouangolodougou. The movement of cattle across boarders for commercial purposes is universal in West Africa. Mobile pastoralism is one mode by which this occurs and is a common way of livelihood in parts of West Africa, including Côte d’Ivoire. Pastoralists migrate from Mali and Burkina Faso to Ferkessédougou and Niakaramadougou in the dry season to graze their animals in the Savannah and return to their homes in the rainy season, sometimes selling their livestock along the way [58]. Aside from mobile pastoralism, cattle are also transported by train or truck from other countries to Bouaké, Yamoussoukro or Agboville to be sold, after which they may be slaughtered or taken to abattoirs in other centres. This translates to many opportunities for parasites to disperse across large distances and ecosystems and may be one reason for the lack of genetic structuring amongst schistosomes from cattle in Côte d’Ivoire. We expected schistosomes from the site in the West (Duekoué) to show some distinction from the other sites, because it is not on the main cattle migration route and because an analysis of schistosomes from humans at the same sites revealed structuring between Duekoué and the other sites [38]. However, no such geographic structuring was apparent in cattle parasites. Possible explanations are as follows. First, parasite mixing in the cattle population may be too intense across the strongly interconnected economic zone from Mali and Burkina Faso to the coastal harbours of Côte d’Ivoire for genetic structuring to arise. Second, the methodological problem of reliably verifying the origin of cattle may have blurred patterns even if they were present. In abattoirs, we sampled only livestock which were reported to be from Côte d’Ivoire, but the reliability of this information is not certain. Animals on farms were reported to be almost exclusively born and kept on farms; however, some uncertainty remains. This potential difference may have distorted our results by blurring geographic patterns and the comparison of miracidia from farm cattle and adult worms from slaughtered cattle.

There was virtually no difference or structuring between male and female populations of schistosomes, which is congruent with their mode of transmission and limited possibilities to modulate their dispersal. In our analysis the genetic diversity of adult schistosomes inside a host individual is comparable to that found across the host population. The slightly lower diversity we found in the few individual cattle in which we analysed many worms can be expected due to sampling imbalance. Our between values were based on a sample size double that of the individual hosts. This might result in enhanced diversity, even if our resampling scheme assured equal group sizes in every resampling round. The key result is, however, that for all measures we analysed, some individual cows harboured as much diversity as the overall population. This finding contrasts with an investigation in Cameroonian cattle and the general pattern across *S. haematobium* and *S. mansoni*, where most of the genetic variation is found within as opposed to between hosts [22, 30]. Our data indicate that–at least in cattle–the definitive host or its behaviour does not reduce the schistosome genetic diversity present in the environment. Moreover, our findings suggest that many schistosomes from few individual cattle can represent the local parasite population in population genetic analyses, since they do not appear to be more closely related than across hosts. However, caution is indicated regarding this theoretical consideration and findings by other researchers [38] who advocate for using a broad host sampling. It must be kept in mind that the same is not true for miracidia, i.e. for studies using live hosts, where sampling many parasites from a few host individuals might miss important components of diversity that are visible only across the host population.

Genetic diversity and population genetic patterns were compared between adult schistosomes and miracidia to assess whether analysis of the two life stages yield comparable results and whether one can be substituted for the other. It appears that miracidia and adult schistosomes from farms and abattoirs, respectively, in the same region harbour indistinguishable genotypes. Especially high levels of inbreeding were seen in the miracidia population compared to adult worms. This trend was visible in the within-host analysis as well when comparing within live cattle to slaughtered cattle. Our findings suggest that while adult worms and miracidia are part of the same gene pool, there are processes that lead to different genetic diversity and inbreeding and that the two life stages cannot simply be combined in analyses, even in the same region.

Transmission dynamics are best studied by collecting parasites from all definitive and intermediate hosts. While we collected adult schistosomes and miracidia from sheep and goats as well as cercariae from snails, the prevalences in these hosts were very low; hence, we were not able to obtain sufficiently large samples to analyse schistosomes from the other hosts [11]. Our study was part of a One Health project [11, 16, 34, 36, 38, 53, 59]. Concurrent studies on human schistosomiasis and the role of pastoral systems show the benefits of a One Health approach by revealing epidemiological trends in livestock, which can inform prevention, control and elimination efforts. Despite high prevalences of schistosomiasis in livestock and the identification of zoonotic disease management as part of the prevention strategy in the current WHO NTD roadmap, there are currently no schistosomiasis surveillance or control programmes for livestock in Africa in general and Côte d’Ivoire more specifically [6, 11, 60, 61]. Hybrids of livestock and human schistosomes highlight the mobility and potential for host expansion of these parasites. Our data suggest that there is no transmission from humans to cattle, while other studies have demonstrated gene flow from cattle to human parasites [12, 21, 38]. Further research is needed to confirm and monitor this situation involving all possible hosts. Aiming to interrupt schistosomiasis transmission in humans and cattle requires that open defecation in the vicinity of water bodies is ceased. Participatory processes with communities could pave the way for socio-culturally acceptable ways of avoiding open defecation through latrine construction. In livestock, the provision of watering places that avoid animals entering the water bodies could reduce transmission, but they must be developed in a locally acceptable way.

## Conclusions

Despite the presence of human and bovine hybrid schistosomes in Ivorian humans, the current study found no evidence of hybrids or *S. curassoni* in cattle. Schistosome populations in cattle from the north and south of Côte d’Ivoire appear to be panmictic. The WHO NTD roadmap [6, 61] advocates for a One Health approach as one of the strategies for controlling NTDs, including schistosomiasis. The combination of the presence of hybrids in humans and the panmictic population of livestock schistosomes suggests a need for a livestock schistosome control programme.

### Supplementary Information

**Additional file 1: Table S1.** ID, life stage, location of collection and results of genotyping and sequencing of all *Schistosoma* samples. **Table S2.** Statistical comparison by linear model of between- versus within-host genetic diversity in Ferkessédougou. **Table S3.** F_ST_ and R_ST_ of populations of *Schistosoma* from within eight cattle hosts. **Fig. S1.** Determination of best K (largest Delta K) for structure analyses.

## Data Availability

The data generated during the current study are available in the NCBI GenBank repository PP312934-3034 and PP313051-54. Microsatellite datasets are available from the corresponding author on reasonable request.

## Abbreviations

A: Alleles
Ar: Rarified allelic richness
best K: Best number of clusters or subpopulations
bp: Base pair
CI: Confidence intervals
*cox1*: Cyclooxygenase 1
DAPC: Discriminant analysis of principal components
dNTPs: Deoxyribonucleotide triphosphate
EDTA: Ethylenediaminetetraacetic acid
F_IS_: Fixation index
FTA: Flinders Technology Associates
H_e_: Expected heterozygosity
H_o_: Observed heterozygosity
*ITS1/2*: Internal transcribed spacers 1 and 2
MCMC: Markov chain Monte Carlo
MgCl_2_: Magnesium chloride
MIRAH: Ministère des Ressources Animales et Halieutiques
NTD: Neglected tropical diseases
PBS: Phosphate-buffered saline
PCR: Polymerase chain reaction
P^HWE^: Probability of deviation from Hardy-Weinberg equilibrium
SNP: Single nucleotide polymorphisms
TBE: Tris–borate-EDTA
TE buffer: Tris-EDTA buffer
Tris-HCl: Tris hydrochloride
WHO: World Health Organization
