# Supplementary material for: Genetic characterization of schistosome species from cattle in Côte d’Ivoire
Source: Parasit Vectors. 2024 Mar 12;17:122. doi: 10.1186/s13071-024-06221-9 (PMC10935785; doi:10.1186/s13071-024-06221-9)
Supplement: Supplementary file 1 — Additional file 1: Table S1. ID, life stage, location of collection and results of genotyping and sequencing of all Schistosoma samples. Table S2. Statistical comparison by linear model of between- versus within-host genetic diversity in Ferkessédougou. Table S3. FST and RST of populations of Schistosoma from within eight cattle hosts. Fig. S1. Determination of best K (largest Delta K) for structure analyses. [file 13071_2024_6221_MOESM1_ESM.docx]

**Additional Material**

**Table S1**. ID, life stage, location of collection and results of genotyping and sequencing of all *Schistosoma* samples analyzed. Adult worms were collected in abattoirs, miracidia from live animals on farms. Sample ID with SA= schistosome adult, with SM=schistosome miracidia; n/a, did not amplify; -, not done.

| Sample ID | Host ID | Sex | Location | Multiplex *cox1* species | *ITS* species |
| --- | --- | --- | --- | --- | --- |
| SA036 | AQ42 | M | Ouangolodougou | *S. bovis* | *S. bovis* |
| SA038 | AQ41 | F | Ouangolodougou | *S. bovis* | *S. bovis* |
| SA039 | AQ41 | M | Ouangolodougou | *S. bovis* | *S. bovis* |
| SA154 | AQ41 | M | Ouangolodougou | *S. bovis* | *S. bovis* |
| SA155 | AQ41 | F | Ouangolodougou | *S. bovis* | - |
| SA156 | AQ41 | M | Ouangolodougou | *S. bovis* | *S. bovis* |
| SA157 | AQ41 | F | Ouangolodougou | *S. bovis* | - |
| SA158 | AQ41 | M | Ouangolodougou | *S. bovis* | *S. bovis* |
| SA159 | AQ41 | F | Ouangolodougou | n/a | *S. bovis* |
| SA160 | AQ41 | F | Ouangolodougou | *S. bovis* | *S. bovis* |
| SA161 | AQ41 | M | Ouangolodougou | *S. bovis* | *S. bovis* |
| SA279 | AQ32 | F | Ouangolodougou | *S. bovis* | - |
| SA280 | AQ32 | M | Ouangolodougou | *S. bovis* | - |
| SA281 | AQ33 | F | Ouangolodougou | *S. bovis* | - |
| SA282 | AQ33 | M | Ouangolodougou | n/a | - |
| SA283 | AQ35 | F | Ouangolodougou | *S. bovis* | - |
| SA284 | AQ35 | M | Ouangolodougou | *S. bovis* | - |
| SA285 | AQ36 | F | Ouangolodougou | *S. bovis* | - |
| SA286 | AQ36 | M | Ouangolodougou | *S. bovis* | - |
| SA287 | AQ37 | F | Ouangolodougou | n/a | - |
| SA288 | AQ37 | M | Ouangolodougou | *S. bovis* | - |
| SA289 | AQ38 | F | Ouangolodougou | *S. bovis* | - |
| SA290 | AQ38 | M | Ouangolodougou | *S. bovis* | - |
| SA291 | AQ39 | F | Ouangolodougou | *S. bovis* | - |
| SA292 | AQ39 | M | Ouangolodougou | *S. bovis* | - |
| SA294 | AQ40 | M | Ouangolodougou | *S. bovis* | - |
| SA390 | AQ34 | M | Ouangolodougou | *S. bovis* | - |
| SA391 | AQ34 | M | Ouangolodougou | *S. bovis* | - |
| SA392 | AQ42 | F | Ouangolodougou | *S. bovis* | - |
| SA393 | AQ42 | M | Ouangolodougou | *S. bovis* | - |
| SA394 | AQ42 | F | Ouangolodougou | *S. bovis* | - |
| SA395 | AQ42 | M | Ouangolodougou | *S. bovis* | - |
| SA398 | AQ40 | M | Ouangolodougou | *S. bovis* | - |
| SA399 | AQ40 | F | Ouangolodougou | *S. bovis* | - |
| SA400 | AQ32 | M | Ouangolodougou | *S. bovis* | - |
| SA003 | AQ10 | M | Ferkessédougou | *S. bovis* | - |
| SA004 | AQ10 | F | Ferkessédougou | *S. bovis* | - |
| SA148 | AQ10 | M | Ferkessédougou | *S. bovis* | *S. bovis* |
| SA149 | AQ10 | M | Ferkessédougou | *S. bovis* | *S. bovis* |
| SA152 | AQ23 | M | Ferkessédougou | *S. bovis* | *S. bovis* |
| SA164 | AQ10 | F | Ferkessédougou | *S. bovis* | *S. bovis* |
| SA165 | AQ10 | F | Ferkessédougou | *S. bovis* | *S. bovis* |
| SA166 | AQ10 | M | Ferkessédougou | *S. bovis* | *S. bovis* |
| SA167 | AQ23 | F | Ferkessédougou | *S. bovis* | *S. bovis* |
| SA168 | AQ23 | F | Ferkessédougou | *S. bovis* | *S. bovis* |
| SA169 | AQ23 | M | Ferkessédougou | *S. bovis* | *S. bovis* |
| SA170 | AQ23 | F | Ferkessédougou | *S. bovis* | *S. bovis* |
| SA171 | AQ23 | M | Ferkessédougou | n/a | *S. bovis* |
| SA174 | AQ10 | F | Ferkessédougou | *S. bovis* | *S. bovis* |
| SA175 | Cfr03 | F | Ferkessédougou | *S. bovis* | - |
| SA176 | Cfr03 | M | Ferkessédougou | *S. bovis* | - |
| SA177 | Cfr08 | F | Ferkessédougou | *S. bovis* | - |
| SA178 | Cfr08 | M | Ferkessédougou | *S. bovis* | - |
| SA179 | Cfr10 | F | Ferkessédougou | *S. bovis* | - |
| SA180 | Cfr10 | M | Ferkessédougou | *S. bovis* | - |
| SA181 | Cfr12 | F | Ferkessédougou | *S. bovis* | - |
| SA182 | Cfr12 | M | Ferkessédougou | *S. bovis* | - |
| SA183 | Cfr13 | F | Ferkessédougou | *S. bovis* | - |
| SA184 | Cfr13 | M | Ferkessédougou | *S. bovis* | - |
| SA185 | Cfr14 | F | Ferkessédougou | *S. bovis* | - |
| SA186 | Cfr14 | M | Ferkessédougou | *S. bovis* | - |
| SA187 | Cfr15 | F | Ferkessédougou | *S. bovis* | - |
| SA188 | Cfr15 | M | Ferkessédougou | *S. bovis* | - |
| SA189 | Cfr16 | F | Ferkessédougou | *S. bovis* | - |
| SA190 | Cfr16 | M | Ferkessédougou | *S. bovis* | - |
| SA191 | Cfr17 | F | Ferkessédougou | *S. bovis* | - |
| SA192 | Cfr17 | M | Ferkessédougou | *S. bovis* | - |
| SA193 | Cfr19 | F | Ferkessédougou | *S. bovis* | - |
| SA194 | Cfr19 | M | Ferkessédougou | *S. bovis* | - |
| SA195 | Cfr20 | F | Ferkessédougou | *S. bovis* | - |
| SA196 | Cfr20 | M | Ferkessédougou | *S. bovis* | - |
| SA197 | Cfr26 | F | Ferkessédougou | *S. bovis* | - |
| SA198 | Cfr26 | M | Ferkessédougou | *S. bovis* | - |
| SA199 | Cfr30 | F | Ferkessédougou | *S. bovis* | - |
| SA200 | Cfr30 | M | Ferkessédougou | *S. bovis* | - |
| SA201 | Cfr33 | F | Ferkessédougou | *S. bovis* | - |
| SA202 | Cfr33 | M | Ferkessédougou | *S. bovis* | - |
| SA203 | Cfr45 | F | Ferkessédougou | *S. bovis* | - |
| SA204 | Cfr45 | M | Ferkessédougou | *S. bovis* | - |
| SA205 | Cfr66 | F | Ferkessédougou | *S. bovis* | - |
| SA206 | Cfr46 | M | Ferkessédougou | *S. bovis* | - |
| SA207 | Cfr49 | F | Ferkessédougou | *S. bovis* | - |
| SA208 | Cfr49 | M | Ferkessédougou | *S. bovis* | - |
| SA210 | Cfr55 | F | Ferkessédougou | *S. bovis* | - |
| SA211 | Cfr55 | M | Ferkessédougou | *S. bovis* | - |
| SA212 | Cfr58 | F | Ferkessédougou | *S. bovis* | - |
| SA213 | Cfr58 | M | Ferkessédougou | *S. bovis* | - |
| SA214 | Cfr60 | F | Ferkessédougou | *S. bovis* | - |
| SA215 | Cfr60 | M | Ferkessédougou | *S. bovis* | - |
| SA216 | Cfr61 | F | Ferkessédougou | *S. bovis* | - |
| SA217 | Cfr61 | M | Ferkessédougou | *S. bovis* | - |
| SA218 | Cfr66 | F | Ferkessédougou | *S. bovis* | - |
| SA219 | Cfr66 | M | Ferkessédougou | *S. bovis* | - |
| SA220 | Cfr72 | F | Ferkessédougou | *S. bovis* | - |
| SA221 | Cfr72 | M | Ferkessédougou | *S. bovis* | - |
| SA222 | Cfr76 | F | Ferkessédougou | *S. bovis* | - |
| SA223 | Cfr76 | M | Ferkessédougou | *S. bovis* | - |
| SA224 | Cfr78 | F | Ferkessédougou | *S. bovis* | - |
| SA225 | Cfr78 | M | Ferkessédougou | *S. bovis* | - |
| SA226 | Cfr80 | F | Ferkessédougou | *S. bovis* | - |
| SA227 | Cfr80 | M | Ferkessédougou | *S. bovis* | - |
| SA228 | Cfr81 | F | Ferkessédougou | *S. bovis* | - |
| SA229 | Cfr81 | M | Ferkessédougou | *S. bovis* | - |
| SA230 | Cfr83 | F | Ferkessédougou | *S. bovis* | - |
| SA231 | Cfr83 | M | Ferkessédougou | *S. bovis* | - |
| SA233 | Cfr84 | F | Ferkessédougou | *S. bovis* | - |
| SA234 | Cfr84 | M | Ferkessédougou | *S. bovis* | - |
| SA235 | Cfr85 | F | Ferkessédougou | n/a | - |
| SA236 | Cfr85 | M | Ferkessédougou | n/a | - |
| SA237 | Cfr87 | F | Ferkessédougou | *S. bovis* | - |
| SA238 | Cfr87 | M | Ferkessédougou | *S. bovis* | - |
| SA239 | Cfr90 | F | Ferkessédougou | n/a | - |
| SA240 | Cfr90 | M | Ferkessédougou | *S. bovis* | - |
| SA241 | Cfr95 | F | Ferkessédougou | n/a | - |
| SA242 | Cfr95 | M | Ferkessédougou | *S. bovis* | - |
| SA243 | Cfr10 | F | Ferkessédougou | *S. bovis* | - |
| SA244 | Cfr10 | M | Ferkessédougou | *S. bovis* | - |
| SA245 | Cfr10 | F | Ferkessédougou | *S. bovis* | - |
| SA246 | Cfr10 | M | Ferkessédougou | *S. bovis* | - |
| SA247 | Cfr10 | F | Ferkessédougou | *S. bovis* | - |
| SA248 | Cfr10 | M | Ferkessédougou | *S. bovis* | - |
| SA249 | Cfr10 | F | Ferkessédougou | *S. bovis* | - |
| SA250 | Cfr10 | M | Ferkessédougou | *S. bovis* | - |
| SA251 | Cfr11 | F | Ferkessédougou | *S. bovis* | - |
| SA252 | Cfr11 | M | Ferkessédougou | *S. bovis* | - |
| SA253 | Cfr11 | F | Ferkessédougou | n/a | - |
| SA254 | Cfr11 | M | Ferkessédougou | *S. bovis* | - |
| SA255 | Cfr11 | M | Ferkessédougou | *S. bovis* | - |
| SA256 | Cfr11 | F | Ferkessédougou | n/a | - |
| SA257 | Cfr11 | M | Ferkessédougou | n/a | - |
| SA258 | Cfr84 | F | Ferkessédougou | *S. bovis* | - |
| SA259 | Cfr84 | M | Ferkessédougou | *S. bovis* | - |
| SA260 | Cfr84 | F | Ferkessédougou | *S. bovis* | - |
| SA261 | Cfr84 | M | Ferkessédougou | n/a | - |
| SA262 | Cfr84 | F | Ferkessédougou | *S. bovis* | - |
| SA263 | Cfr84 | M | Ferkessédougou | n/a | - |
| SA264 | Cfr84 | F | Ferkessédougou | n/a | - |
| SA265 | Cfr84 | M | Ferkessédougou | *S. bovis* | - |
| SA266 | Cfr84 | F | Ferkessédougou | *S. bovis* | - |
| SA267 | Cfr84 | M | Ferkessédougou | n/a | - |
| SA268 | Cfr84 | F | Ferkessédougou | *S. bovis* | - |
| SA269 | Cfr84 | M | Ferkessédougou | *S. bovis* | - |
| SA270 | Cfr84 | F | Ferkessédougou | *S. bovis* | - |
| SA271 | Cfr84 | M | Ferkessédougou | *S. bovis* | - |
| SA272 | Cfr84 | F | Ferkessédougou | n/a | - |
| SA273 | Cfr84 | M | Ferkessédougou | *S. bovis* | - |
| SA274 | Cfr84 | F | Ferkessédougou | *S. bovis* | - |
| SA275 | Cfr84 | M | Ferkessédougou | *S. bovis* | - |
| SA276 | Cfr84 | M | Ferkessédougou | *S. bovis* | - |
| SA277 | Cfr84 | M | Ferkessédougou | *S. bovis* | - |
| SA278 | Cfr84 | M | Ferkessédougou | *S. bovis* | - |
| SA295 | CFr78 | F | Ferkessédougou | *S. bovis* | - |
| SA296 | CFr78 | M | Ferkessédougou | *S. bovis* | - |
| SA297 | CFr78 | F | Ferkessédougou | *S. bovis* | - |
| SA298 | CFr78 | M | Ferkessédougou | *S. bovis* | - |
| SA299 | CFr78 | F | Ferkessédougou | *S. bovis* | - |
| SA300 | CFr78 | M | Ferkessédougou | *S. bovis* | - |
| SA301 | CFr78 | F | Ferkessédougou | *S. bovis* | - |
| SA302 | CFr78 | M | Ferkessédougou | *S. bovis* | - |
| SA303 | CFr78 | F | Ferkessédougou | *S. bovis* | - |
| SA304 | CFr78 | M | Ferkessédougou | *S. bovis* | - |
| SA305 | CFr78 | F | Ferkessédougou | *S. bovis* | - |
| SA306 | CFr78 | M | Ferkessédougou | *S. bovis* | - |
| SA307 | CFr78 | F | Ferkessédougou | *S. bovis* | - |
| SA308 | CFr78 | M | Ferkessédougou | *S. bovis* | - |
| SA309 | CFr78 | F | Ferkessédougou | *S. bovis* | - |
| SA310 | CFr78 | M | Ferkessédougou | *S. bovis* | - |
| SA311 | CFr78 | F | Ferkessédougou | *S. bovis* | - |
| SA312 | CFr78 | M | Ferkessédougou | *S. bovis* | - |
| SA313 | CFr20 | F | Ferkessédougou | *S. bovis* | - |
| SA314 | CFr20 | M | Ferkessédougou | *S. bovis* | - |
| SA315 | CFr20 | F | Ferkessédougou | *S. bovis* | - |
| SA316 | CFr20 | M | Ferkessédougou | *S. bovis* | - |
| SA317 | CFr20 | F | Ferkessédougou | *S. bovis* | - |
| SA318 | CFr20 | M | Ferkessédougou | *S. bovis* | - |
| SA319 | CFr20 | F | Ferkessédougou | *S. bovis* | - |
| SA320 | CFr20 | M | Ferkessédougou | *S. bovis* | - |
| SA321 | CFr20 | F | Ferkessédougou | *S. bovis* | - |
| SA322 | CFr20 | M | Ferkessédougou | *S. bovis* | - |
| SA323 | CFr20 | F | Ferkessédougou | *S. bovis* | - |
| SA324 | CFr20 | M | Ferkessédougou | *S. bovis* | - |
| SA325 | CFr20 | F | Ferkessédougou | *S. bovis* | - |
| SA326 | CFr20 | M | Ferkessédougou | *S. bovis* | - |
| SA327 | CFr20 | F | Ferkessédougou | *S. bovis* | - |
| SA328 | CFr20 | M | Ferkessédougou | *S. bovis* | - |
| SA329 | CFr20 | F | Ferkessédougou | *S. bovis* | - |
| SA330 | CFr20 | M | Ferkessédougou | *S. bovis* | *-* |
| SM008 | BV34 |  | Ferkessédougou | *S. bovis* | *S. bovis* |
| SM009 | BV23 |  | Ferkessédougou | *S. bovis* | *S. bovis* |
| SM010 | BV04 |  | Ferkessédougou | *S. bovis* | *S. bovis* |
| SM011 | BV21 |  | Ferkessédougou | *S. bovis* | *S. bovis* |
| SM061 | BV34 |  | Ferkessédougou | *S. bovis* | - |
| SM062 | BV34 |  | Ferkessédougou | *S. bovis* | - |
| SM065 | BV34 |  | Ferkessédougou | *S. bovis* | - |
| SM066 | BV34 |  | Ferkessédougou | *S. bovis* | - |
| SM067 | 168 |  | Ferkessédougou | *S. bovis* | - |
| SM068 | 180 |  | Ferkessédougou | *S. bovis* | - |
| SM069 | 195 |  | Ferkessédougou | *S. bovis* | - |
| SM070 | 232 |  | Ferkessédougou | *S. bovis* | - |
| SM071 | 236 |  | Ferkessédougou | *S. bovis* | - |
| SM072 | 264 |  | Ferkessédougou | *S. bovis* | - |
| SM073 | 185 |  | Ferkessédougou | *S. bovis* | - |
| SM074 | 225 |  | Ferkessédougou | *S. bovis* | - |
| SM075 | 233 |  | Ferkessédougou | *S. bovis* | - |
| SM076 | 238 |  | Ferkessédougou | *S. bovis* | - |
| SM078 | 242 |  | Ferkessédougou | *S. bovis* | - |
| SM079 | 245 |  | Ferkessédougou | *S. bovis* | - |
| SM082 | 249 |  | Ferkessédougou | *S. bovis* | - |
| SM083 | 250 |  | Ferkessédougou | *S. bovis* | - |
| SM084 | 252 |  | Ferkessédougou | *S. bovis* | - |
| SM085 | 257 |  | Ferkessédougou | *S. bovis* | - |
| SM086 | 260 |  | Ferkessédougou | *S. bovis* | - |
| SM087 | 261 |  | Ferkessédougou | *S. bovis* | - |
| SM088 | 262 |  | Ferkessédougou | *S. bovis* | - |
| SM089 | 263 |  | Ferkessédougou | *S. bovis* | - |
| SM090 | 267 |  | Ferkessédougou | *S. bovis* | - |
| SM091 | 268 |  | Ferkessédougou | *S. bovis* | - |
| SM092 | 270 |  | Ferkessédougou | *S. bovis* | - |
| SM105 | 195 |  | Ferkessédougou | n/a | - |
| SM106 | 195 |  | Ferkessédougou | *S. bovis* | - |
| SM107 | 195 |  | Ferkessédougou | *S. bovis* | - |
| SM108 | 195 |  | Ferkessédougou | *S. bovis* | - |
| SM109 | 195 |  | Ferkessédougou | *S. bovis* | - |
| SM110 | 195 |  | Ferkessédougou | *S. bovis* | - |
| SM111 | 195 |  | Ferkessédougou | *S. bovis* | - |
| SM113 | 195 |  | Ferkessédougou | *S. bovis* | - |
| SM114 | 195 |  | Ferkessédougou | *S. bovis* | - |
| SM115 | 195 |  | Ferkessédougou | *S. bovis* | - |
| SM116 | 195 |  | Ferkessédougou | *S. bovis* | - |
| SM117 | 195 |  | Ferkessédougou | *S. bovis* | - |
| SM118 | 195 |  | Ferkessédougou | *S. bovis* | - |
| SM119 | 195 |  | Ferkessédougou | *S. bovis* | - |
| SM120 | 195 |  | Ferkessédougou | n/a | - |
| SM122 | 195 |  | Ferkessédougou | *S. bovis* | - |
| SM123 | BV34 |  | Ferkessédougou | *S. bovis* | - |
| SM124 | BV34 |  | Ferkessédougou | *S. bovis* | - |
| SM125 | BV34 |  | Ferkessédougou | *S. bovis* | - |
| SM126 | BV34 |  | Ferkessédougou | *S. bovis* | - |
| SM127 | BV34 |  | Ferkessédougou | *S. bovis* | - |
| SM129 | BV34 |  | Ferkessédougou | *S. bovis* | - |
| SM130 | BV34 |  | Ferkessédougou | *S. bovis* | - |
| SM131 | BV34 |  | Ferkessédougou | *S. bovis* | - |
| SM132 | BV34 |  | Ferkessédougou | *S. bovis* | - |
| SM134 | BV34 |  | Ferkessédougou | *S. bovis* | - |
| SM135 | BV34 |  | Ferkessédougou | *S. bovis* | - |
| SM136 | BV34 |  | Ferkessédougou | *S. bovis* | - |
| SM137 | 236 |  | Ferkessédougou | *S. bovis* | - |
| SM138 | 236 |  | Ferkessédougou | *S. bovis* | - |
| SM139 | 236 |  | Ferkessédougou | *S. bovis* | - |
| SM140 | 236 |  | Ferkessédougou | *S. bovis* | - |
| SM141 | 236 |  | Ferkessédougou | *S. bovis* | - |
| SM142 | 236 |  | Ferkessédougou | *S. bovis* | - |
| SM143 | 236 |  | Ferkessédougou | *S. bovis* | - |
| SM144 | 236 |  | Ferkessédougou | *S. bovis* | - |
| SM145 | 236 |  | Ferkessédougou | *S. bovis* | - |
| SM146 | 236 |  | Ferkessédougou | *S. bovis* | - |
| SM147 | 236 |  | Ferkessédougou | *S. bovis* | - |
| SM150 | BV04 |  | Ferkessédougou | *S. bovis* | - |
| SM151 | BV04 |  | Ferkessédougou | *S. bovis* | - |
| SM152 | BV04 |  | Ferkessédougou | *S. bovis* | - |
| SM153 | BV04 |  | Ferkessédougou | *S. bovis* | - |
| SM155 | BV04 |  | Ferkessédougou | *S. bovis* | - |
| SM156 | BV04 |  | Ferkessédougou | *S. bovis* | - |
| SM158 | BV04 |  | Ferkessédougou | *S. bovis* | - |
| SM159 | BV04 |  | Ferkessédougou | *S. bovis* | - |
| SM160 | BV04 |  | Ferkessédougou | *S. bovis* | - |
| SM162 | 232 |  | Ferkessédougou | *S. bovis* | - |
| SM163 | 232 |  | Ferkessédougou | *S. bovis* | - |
| SM164 | 232 |  | Ferkessédougou | *S. bovis* | - |
| SM165 | 232 |  | Ferkessédougou | *S. bovis* | - |
| SM166 | 232 |  | Ferkessédougou | *S. bovis* | - |
| SM167 | 232 |  | Ferkessédougou | *S. bovis* | - |
| SM168 | 232 |  | Ferkessédougou | *S. bovis* | - |
| SM169 | 232 |  | Ferkessédougou | *S. bovis* | - |
| SM170 | 232 |  | Ferkessédougou | *S. bovis* | - |
| SM188 | BV21 |  | Ferkessédougou | *S. bovis* | - |
| SM189 | 247 |  | Ferkessédougou | *S. bovis* | - |
| SM190 | 248 |  | Ferkessédougou | *S. bovis* | - |
| SM191 | 247 |  | Ferkessédougou | *S. bovis* | - |
| SM192 | 247 |  | Ferkessédougou | *S. bovis* | - |
| SM193 | 021 |  | Ferkessédougou | *S. bovis* | - |
| SM194 | 241 |  | Ferkessédougou | *S. bovis* | - |
| SM196 | 242 |  | Ferkessédougou | *S. bovis* | - |
| SM199 | 250 |  | Ferkessédougou | *S. bovis* | - |
| SM200 | 262 |  | Ferkessédougou | *S. bovis* | - |
| SA358 | OD83 | M | Odienné | *S. bovis* | - |
| SA359 | OD83 | F | Odienné | *S. bovis* | - |
| SA386 | Bd25 | M | Bondoukou | *S. bovis* | - |
| SA387 | Bd25 | F | Bondoukou | *S. bovis* | - |
| SA388 | Bd25 | M | Bondoukou | *S. bovis* | - |
| SA389 | Bd25 | F | Bondoukou | *S. bovis* | - |
| SA375 | AB28 | M | Abengourou | *S. bovis* | - |
| SA376 | AB28 | F | Abengourou | *S. bovis* | - |
| SA377 | AB28 | M | Abengourou | *S. bovis* | - |
| SA378 | AB28 | F | Abengourou | *S. bovis* | - |
| SA379 | AB28 | M | Abengourou | *S. bovis* | - |
| SA381 | AB28 | M | Abengourou | *S. bovis* | - |
| SA382 | AB03 | M | Abengourou | *S. bovis* | - |
| SA383 | AB03 | F | Abengourou | *S. bovis* | - |
| SA384 | AB39 | M | Abengourou | *S. bovis* | - |
| SA385 | AB39 | F | Abengourou | *S. bovis* | - |
| SA007 | AL46 | M | Duekoué | *S. bovis* | *S. bovis* |
| SA008 | AL46 | F | Duekoué | *S. bovis* | *S. bovis* |
| SA032 | AM15 | M | Duekoué | *S. bovis* | *S. bovis* |
| SA033 | AM15 | F | Duekoué | *S. bovis* | *S. bovis* |
| SA034 | AL46 | F | Duekoué | *S. bovis* | *S. bovis* |
| SA035 | AL46 | M | Duekoué | *S. bovis* | *S. bovis* |
| SA048 | AL46 | M | Duekoué | *S. bovis* | *S. bovis* |
| SA049 | AL46 | F | Duekoué | *S. bovis* | *S. bovis* |
| SA050 | AM15 | M | Duekoué | *S. bovis* | - |
| SA051 | AM15 | M | Duekoué | *S. bovis* | - |
| SA120 | AL46 | M | Duekoué | *S. bovis* | *S. bovis* |
| SA121 | AL46 | F | Duekoué | n/a | *S. bovis* |
| SA122 | AL46 | M | Duekoué | *S. bovis* | *S. bovis* |
| SA123 | AL46 | F | Duekoué | n/a | *S. bovis* |
| SA124 | AL46 | M | Duekoué | *S. bovis* | *S. bovis* |
| SA331 | DUB066 | M | Duekoué | *S. bovis* | - |
| SA332 | DUB066 | F | Duekoué | *S. bovis* | - |
| SA333 | DUB066 | M | Duekoué | *S. bovis* | - |
| SA334 | DUB066 | F | Duekoué | *S. bovis* | - |
| SA335 | DUB066 | M | Duekoué | *S. bovis* | - |
| SA336 | DUB066 | F | Duekoué | *S. bovis* | - |
| SA337 | DUB066 | M | Duekoué | *S. bovis* | - |
| SA338 | DUB066 | F | Duekoué | *S. bovis* | - |
| SA339 | DUB066 | M | Duekoué | *S. bovis* | - |
| SA340 | DUB066 | F | Duekoué | *S. bovis* | - |
| SA341 | DUB066 | M | Duekoué | *S. bovis* | - |
| SA342 | DUB066 | F | Duekoué | *S. bovis* | - |
| SA343 | DUB066 | M | Duekoué | *S. bovis* | - |
| SA344 | DUB066 | F | Duekoué | *S. bovis* | - |
| SA345 | DUB086 | M | Duekoué | *S. bovis* | - |
| SA346 | DUB086 | F | Duekoué | *S. bovis* | - |
| SA347 | DUB089 | M | Duekoué | *S. bovis* | - |
| SA348 | DUB089 | F | Duekoué | *S. bovis* | - |
| SA349 | DUB089 | M | Duekoué | *S. bovis* | - |
| SA350 | DUB089 | F | Duekoué | *S. bovis* | - |
| SA351 | DUB089 | M | Duekoué | *S. bovis* | - |
| SA352 | DUB089 | F | Duekoué | *S. bovis* | - |
| SA353 | DUB004 | M | Duekoué | *S. bovis* | - |
| SA354 | DUB004 | F | Duekoué | *S. bovis* | - |
| SA355 | DUB013 | M | Duekoué | *S. bovis* | - |
| SA356 | DUB013 | F | Duekoué | *S. bovis* | - |
| SA357 | DUB086 | F | Duekoué | *S. bovis* | - |
| SA360 | DUB066 | M | Duekoué | *S. bovis* | - |
| SA361 | DUB066 | M | Duekoué | *S. bovis* | - |
| SA362 | DUB066 | M | Duekoué | *S. bovis* | - |
| SA363 | DUB066 | M | Duekoué | *S. bovis* | - |
| SA364 | DUB066 | M | Duekoué | *S. bovis* | - |
| SA365 | DUB066 | M | Duekoué | *S. bovis* | - |
| SA366 | DUB066 | M | Duekoué | *S. bovis* | - |
| SA367 | DUB066 | M | Duekoué | *S. bovis* | - |
| SA368 | DUB066 | M | Duekoué | *S. bovis* | - |
| SA369 | DUB066 | M | Duekoué | *S. bovis* | - |
| SA370 | DUB066 | M | Duekoué | *S. bovis* | - |
| SA371 | DUB086 | M | Duekoué | *S. bovis* | - |
| SA372 | DUB086 | M | Duekoué | *S. bovis* | - |
| SA373 | DUB086 | M | Duekoué | *S. bovis* | - |
| SA374 | DUB086 | M | Duekoué | *S. bovis* | - |
| SA019 | AN04 | M | Agboville | *S. bovis* | - |
| SA020 | AN04 | F | Agboville | *S. bovis* | - |
| SA024 | AN08 | F | Agboville | *S. bovis* | - |
| SA025 | AN08 | M | Agboville | *S. bovis* | - |
| SA052 | AN08 | M | Agboville | *S. bovis* | *S. bovis* |
| SA053 | AN08 | F | Agboville | *S. bovis* | *S. bovis* |
| SA054 | AN31 | M | Agboville | *S. bovis* | *S. bovis* |
| SA055 | AN31 | F | Agboville | *S. bovis* | *S. bovis* |
| SA056 | AN20 | F | Agboville | *S. bovis* | *S. bovis* |
| SA057 | AN20 | M | Agboville | *S. bovis* | *S. bovis* |
| SA125 | AN34 | M | Agboville | *S. bovis* | - |
| SA126 | AN34 | F | Agboville | n/a | - |
| SA127 | AN34 | M | Agboville | *S. bovis* | *S. bovis* |
| SA128 | AN34 | F | Agboville | *S. bovis* | *S. bovis* |
| SA129 | AP04 | F | Agboville | *S. bovis* | - |
| SA130 | AP04 | M | Agboville | n/a | - |
| SA131 | AP04 | F | Agboville | n/a | *S. bovis* |
| SA132 | AP04 | M | Agboville | *S. bovis* | *S. bovis* |
| SA139 | AN08 | M | Agboville | *S. bovis* | - |
| SA140 | AN08 | F | Agboville | *S. bovis* | - |
| SA143 | AN34 | F | Agboville | *S. bovis* | - |
| SA144 | AN34 | M | Agboville | *S. bovis* | - |
| SA145 | AM36 | M | Agboville | *S. bovis* | - |
| SA146 | AM36 | M | Agboville | *S. bovis* | - |
| SA001 | AK09 | M | Sikensi | *S. bovis* | - |
| SA002 | AK09 | F | Sikensi | *S. bovis* | - |
| SA009 | AH48 | F | Sikensi | *S. bovis* | - |
| SA010 | AH48 | M | Sikensi | *S. bovis* | *S. bovis* |
| SA011 | AH49 | M | Sikensi | *S. bovis* | *S. bovis* |
| SA012 | AH49 | F | Sikensi | *S. bovis* | - |
| SA015 | AK09 | F | Sikensi | *S. bovis* | - |
| SA016 | AK09 | M | Sikensi | *S. bovis* | *S. bovis* |
| SA017 | AK15 | F | Sikensi | *S. bovis* | - |
| SA018 | AK15 | M | Sikensi | *S. bovis* | *S. bovis* |
| SA044 | AH48 | F | Sikensi | *S. bovis* | *S. bovis* |
| SA045 | AH48 | M | Sikensi | *S. bovis* | - |
| SA046 | AH49 | F | Sikensi | *S. bovis* | *S. bovis* |
| SA047 | AH49 | M | Sikensi | *S. bovis* | *S. bovis* |
| SA065 | AK06 | M | Sikensi | *S. bovis* | *S. bovis* |
| SA082 | AK15 | F | Sikensi | *S. bovis* | - |
| SA083 | AK15 | M | Sikensi | *S. bovis* | - |
| SA090 | AH31 | M | Sikensi | *S. bovis* | - |
| SA091 | AH31 | F | Sikensi | *S. bovis* | - |
| SA096 | AH31 | F | Sikensi | *S. bovis* | *S. bovis* |
| SA097 | AH31 | M | Sikensi | *S. bovis* | *S. bovis* |
| SA105 | AH48 | M | Sikensi | *S. bovis* | - |
| SA106 | AH49 | F | Sikensi | *S. bovis* | *S. bovis* |
| SA107 | AH49 | M | Sikensi | *S. bovis* | *S. bovis* |
| SA108 | AH49 | M | Sikensi | *S. bovis* | *S. bovis* |
| SA109 | AH49 | F | Sikensi | *S. bovis* | *S. bovis* |

**Table S2.** Statistical comparison by linear model of between versus within host genetic diversity in Ferkessédougou.

Overall test B vs. W1 B vs. W2 B vs. W3

Measure *Df F p t p t p t p*

H_e_ 3 5.03 0.0025 -3.53 0.0006 -1.136 0.2581 -2.291 0.0235

H_o_ 3 5.45 0.0014 -1.056 0.2928 -1.44 0.1520 3.16 0.0020

A_r_ 3 10.45 <0.0001 -0.52 0.6044 -2.65 0.0090 -5.29 <0.0001

F_IS_ 3 4.08 0.0083 -1.18 0.2391 1.05 0.2981 -2.9 0.0039

Test statistics of the overall test are reported as well as the pairwise contrasts between the “between group” (B, samples from across the host population) and the three cattle hosts of the “within group” (W1-3). A_r_, mean allelic richness; H_o_, mean observed heterozygosity; H_e_, mean estimated heterozygosity; F_IS_, fixation index.

**Table S3**. F_ST_ (above diagonal) and R_ST_ (below diagonal) of populations of *Schistosoma* from within eight cattle hosts.

|  | Cfr20 | Cfr78 | Cfr84 | 195 | 232 | 236 | BV34 | BV04 |
| --- | --- | --- | --- | --- | --- | --- | --- | --- |
| Cfr20 (flukes) |  | -0.003 | 0.019 | *0.011* | *0.016* | *0.011* | *-0.002* | *0.047* |
| Cfr78 (flukes) | -0.014 |  | 0.017 | *0.023* | *0.010* | *0.008* | *-0.002* | *0.030* |
| Cfr84 (flukes) | 0.022 | 0.041 |  | *0.043* | *0.055* | *0.038* | *0.019* | *0.053* |
| 195 (miracidia) | *0.000* | *0.006* | *0.011* |  | 0.044 | 0.039 | 0.017 | 0.044 |
| 232 (miracidia) | *-0.028* | *-0.013* | *-0.005* | 0.006 |  | 0.028 | 0.039 | 0.034 |
| 236 (miracidia) | *-0.006* | *0.018* | *0.000* | 0.003 | -0.023 |  | 0.029 | 0.063 |
| BV34 (miracidia) | *-0.011* | *0.000* | *0.048* | 0.026 | -0.030 | 0.003 |  | 0.033 |
| BV04 (miracidia) | *-0.002* | *0.037* | *0.017* | 0.013 | -0.039 | -0.016 | -0.022 |  |

Miracidia from live cattle from farms, flukes from slaughtered cattle from abattoirs in Ferkessédougou, Côte d’Ivoire. Values between life-stages are in italics.

**Figure S1**. Determination of Best K (largest Delta K) for Structure analyses using Evanno’s method (A) flukes from six sampling sites across Côte d’Ivoire, (B) male versus female flukes, (C) flukes compared to miracidia, one miracidia or fluke couple per host (D) within host analysis comparing miracidia from five live cattle and flukes from three slaughtered cattle.
